# A self-organizing model of perisaccadic visual receptive field dynamics in primate visual and oculomotor system

**DOI:** 10.3389/fncom.2015.00017

**Published:** 2015-02-11

**Authors:** Bedeho M. W. Mender, Simon M. Stringer

**Affiliations:** Department of Experimental Psychology, Centre for Theoretical Neuroscience and Artificial Intelligence, University of OxfordOxford, UK

**Keywords:** perisaccadic, remapping, model, self-organization, LIP, FEF, SC, V3

## Abstract

We propose and examine a model for how perisaccadic visual receptive field dynamics, observed in a range of primate brain areas such as LIP, FEF, SC, V3, V3A, V2, and V1, may develop through a biologically plausible process of unsupervised visually guided learning. These dynamics are associated with remapping, which is the phenomenon where receptive fields anticipate the consequences of saccadic eye movements. We find that a neural network model using a local associative synaptic learning rule, when exposed to visual scenes in conjunction with saccades, can account for a range of associated phenomena. In particular, our model demonstrates predictive and pre-saccadic remapping, responsiveness shifts around the time of saccades, and remapping from multiple directions.

## 1. Introduction

A salient characteristic of visual perception in a natural environment is that, despite the high frequency of saccadic eye movements performed toward potentially interesting objects, our subjective visual experience is that of a continuous examination of a stationary environment. However, since the latency between the retina and higher order visual areas is anywhere between 60 and 100 ms, our visual system should be expected to be incongruent with the visual world for a significant fraction of the time due to the high frequency of saccadic eye movements. In light of this, there has been a great deal of debate about how the brain integrates visual percepts across saccades (Melcher and Colby, 2008). A leading hypothesis dates back to the early work of von Helmholtz and Southall (1924), who suggested that internal monitoring of impending eye movement signals can drive an anticipatory integration mechanism across saccades.

The neurophysiological phenomena of predictive response and trace response truncation, found in primate areas such as LIP (Duhamel et al., 1992), SC (Walker et al., 1995), FEF (Umeno and Goldberg, 1997, 2001), and V3 (Nakamura and Colby, 2002), have later been suggested as the neural basis for this mechanism (Melcher and Colby, 2008). This is because they anticipate the visual consequences of impending eye movements at latencies much lower than in pure fixation tasks. A predictive response will reduce the period of incongruence in the activity of a neuron when its classical receptive field is shifted to the location of some salient stimulus. While trace response truncation will do the same for a neuron which has its classical receptive field shifted away from the location of a salient stimulus.

A number of models accounting for how a neural visual representation could be updated across eye movements have been proposed over the past two decades.

Droulez and Berthoz (1991) presented, for the first time, a neural network model that demonstrated how a dynamically updated eye-centered visual representation could provide the neural substrate for the findings in Mays and Sparks (1980). They showed how a short-term memory map updated continuously by an eye velocity signal could maintain a retinotopically accurate representation of a visual scene. However, the continuous shifting of a neural activity packet based on eye velocity is not compatible with the finding that remapping occurs over a range of neural latencies across a population of neurons, and frequently even prior to saccade onset.

Krommenhoek et al. (1993) investigated through modeling how the intermediate motor layers of the SC could incorporate eye position signals to produce the spatially accurate motor error signal. They found that two different neural network models, based on supervised learning, could achieve this by combining the retinal location of a visual target with the eye position at the time of saccade initiation.

Quaia et al. (1998) presented a model attempting to reproduce the most prominent features of the perisaccadic remapping neurophysiology in LIP, FEF and SC. They also proposed that the ability to remap the activity associated with an extinguished visual saccade target was a possible solution to guiding spatially accurate saccades in the classic double-step saccade paradigm, thereby alleviating the need for a head-centered map. This model shares broad similarities with the work in this paper, however it critically depended on implausibly precise hardwiring at the dendrite level.

The distinction between the model proposed in this paper, and all previous models, is that they do not explore how such neural representations could self-organize in a biolgoically plausible manner. All depend on either explicit synaptic hard wiring, or some form of supervised error-correction learning algorithm.

The model developed here aims to explain the visually-guided development of different kinds of receptive field phenomena observed around the time of saccades in a number of visual and saccade related brain areas, in particular LIP (Duhamel et al., 1992), SC (Walker et al., 1995), FEF (Umeno and Goldberg, 1997, 2001), and V3 (Nakamura and Colby, 2002). The primary phenomena to be modeled were as follows:

- *Predictive Remapping*. The saccade aligned latency of the response of a visual neuron to a saccade bringing a visual stimulus into its classical receptive field is less than the latency in a visual onset fixation task (Duhamel et al., 1992; Walker et al., 1995; Umeno and Goldberg, 1997; Nakamura and Colby, 2002).
- *Pre-saccadic Remapping*. Predictive remapping which begins prior to saccade onset (Duhamel et al., 1992; Walker et al., 1995; Umeno and Goldberg, 1997; Nakamura and Colby, 2002).
- *Trace Remapping*. The response of a visual neuron to a saccade that brings the site of a recently extinguished stimulus into the neuron's classical receptive field (Goldberg and Bruce, 1990; Duhamel et al., 1992; Umeno and Goldberg, 2001).
- *Responsiveness Shift*. Around the time of a saccade, the responsiveness of a neuron gradually declines to stimuli flashed in the pre-saccadic location of its visual receptive field, and gradually increases to stimuli flashed in the post-saccadic location of its visual receptive field (Kusunoki and Goldberg, 2003).
- *Spatially Independent Remapping*. Individual neurons can remap activity from multiple different spatial locations and directions in terms of both strength and latency (Heiser et al., 2005).

## 2. Materials and methods

### 2.1. Hypothesis

It is hypothesized that the phenomena of predictive remapping, pre-saccadic remapping, trace remapping, responsiveness shift and spatially independent remapping may develop through the following biologically plausible process of visually-guided learning.

We assume that the eyes tend to move more rapidly than the head, so that visual stimuli remain stationary with respect to the head during saccades.

Consider the simplest situation in which a saccade shifts a visual stimulus from its pre-saccadic retinal location to a post-saccadic retinal location. During the saccade, some visual neurons will encode the pre-saccadic retinal location of the stimulus. These neurons will be distributed acoss a number of areas of the primate visual system. At the same time, other saccade neurons simultaneously encode the retinotopic target location of the saccade. Again, these saccade neurons may also be distributed across different brain areas. Both types of neurons may send efferent projections that converge onto a competitive population of postsynaptic combination neurons, or perhaps multiple such populations. Individual combination neurons learn to encode particular combinations of pre-saccadic stimulus location and saccade target location. After learning, combination neurons require simultaneous signals from both visual and saccade input neurons in order to fire, and so only become active during saccades.

Visual signals from the retina travel through successive layers of the visual system, as well as back and forth across recurrent connections within layers. This results in neurons in different visual areas firing with different response latencies to stimuli entering their receptive fields. Furthermore, some visual neurons may be kept active some time after a visual stimulus is removed due to activity circulating around local recurrent loops within and between layers leading to attractor states. During a saccade, these effects may ensure that some visual neurons representing the pre-saccadic retinal location of a stimulus are able to maintain their activity for a brief period after the saccade when the stimulus has in fact shifted to the post-saccadic retinal location. These activity delays will then be passed on to the combination neurons representing a combination of pre-saccadic stimulus location and saccade target location. These combination neurons will also be active after the saccade when other visual remapping neurons with shorter response latencies are encoding the post-saccadic retinal location of the stimulus. In this case, associative learning may strengthen the connections from the combination neurons to the remapping neurons representing the corresponding post-saccadic stimulus location.

Then, after this initial phase of visually guided learning, the presence of a stimulus in a pre-saccadic retinal location combined with a saccade is sufficient to activate the corresponding combination neuron, which in turn fires the remapping neurons representing the post-saccadic stimulus location. This remapping will occur even if the stimulus is extinguished before the saccade brings the stimulus into the receptive field of the relevant remapping neurons tuned to what would have been the post-saccadic stimulus location. More generally, the basic mechanism should be able to account for predictive remapping, pre-saccadic remapping, trace remapping, responsiveness shift and spatially independent remapping.

The basic mechanism may be realized in a very wide variety of different network architectures with multiple layers, feedforward and feedback connections between layers, and recurrent connections within layers. There are also various options for implementing the dynamics of individual neurons and synapses. We therefore demonstrate how the core hypothesis can work in the simplest network architecture embodying these principles.

**Figure 1:**
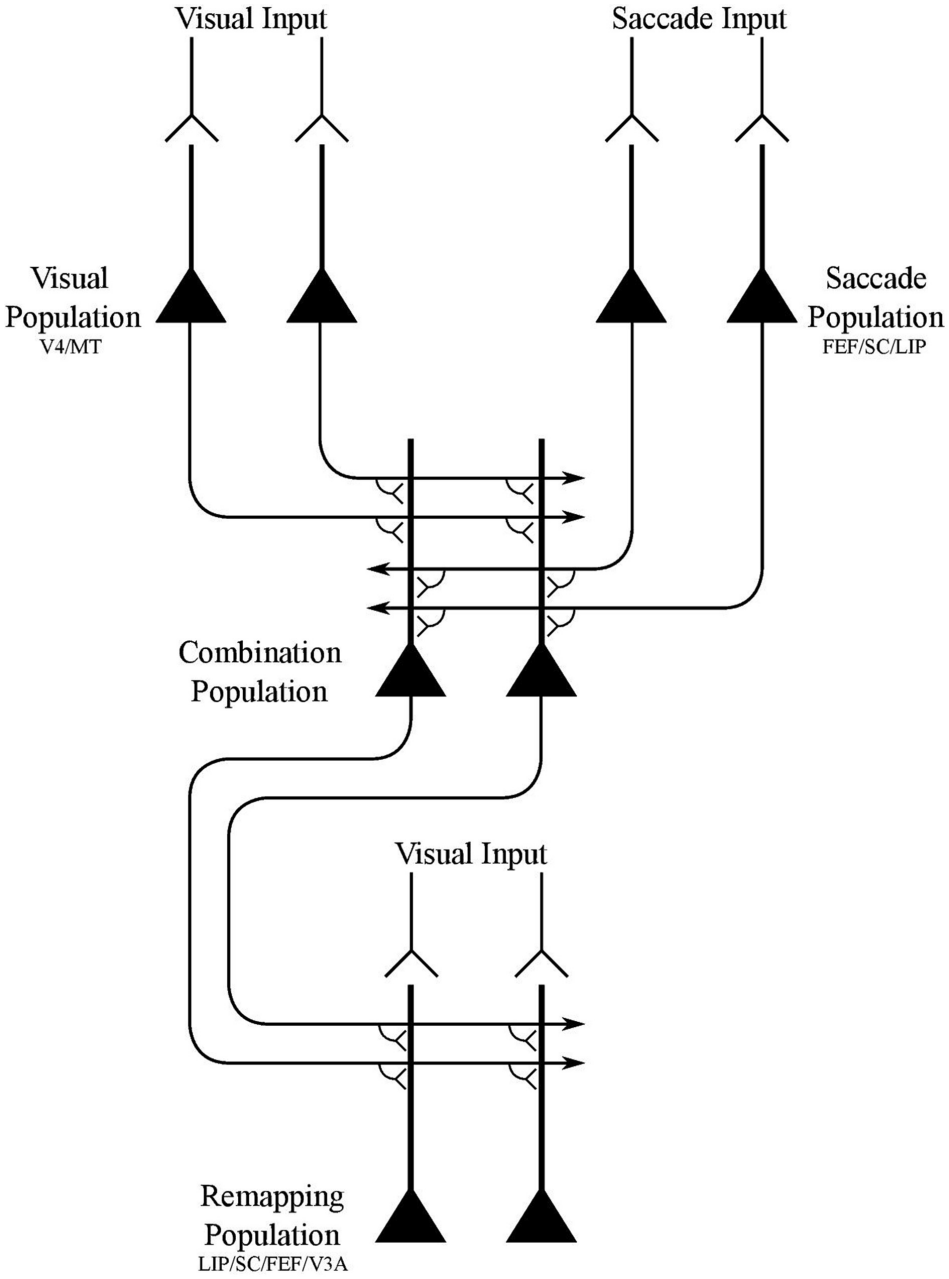
**Network architecture of model**.

In this paper, we demonstrate that the network architecture shown in Figure 1 is able to develop the receptive field dynamics described above through the proposed process of visually guided learning. The components of the model are not intended to be tightly related to specific brain areas, but instead provide the simplest network architecture that is capable of implementing the hypothesized mechanism. Nevertheless, it is possible to make some loose associations between the model components and particular brain areas based on the neuronal response properties reported in these areas, as shown in Figure 1. The model components are as follows:

- *Visual population*. There is a population of visual neurons that represent the retinotopic locations of visual targets. These neurons continue to fire for a short period after the saccade, and thus encode a memory trace of the pre-saccadic visual scene for some time after saccade onset and stimulus offset. These neurons represent a visual map of the stimuli in a scene.
- *Saccade population*. There is a population of eye-centered saccade neurons, which in some perisaccadic time interval encode the retinotopic target location of the impending saccade. These neurons also continue to fire for a short period after the saccade, thus implementing a memory trace of the target location of the last saccade.
- *Combination population*. There is an intermediate population of combination neurons. These neurons receive diluted plastic synaptic connections from the visual population and saccade population, which self-organize during visually-guided learning. Each combination neuron learns to encode a particular combination of the retinotopic location of a visual target and retinotopic target location of the impending saccade. The combination neurons then send efferent connections to all neurons in the remapping population.
- *Remapping population*. There is a population of visual remapping neurons that represent the retinotopic locations of visual targets, and which learn to display the perisaccadic receptive field phenomena of predictive remapping, pre-saccadic remapping, trace remapping and responsiveness shift. The remapping neurons receive plastic synaptic connections from the combination population, which self-organize during visually-guided learning. Each remapping neuron is also driven by three different visual input signals. Specifically, there is a phasic input component that gives rise to a localized burst of activity peaking after a few tens of milliseconds, an underlying tonic input component that keeps the neuron active while the stimulus is present, and a memory trace component that lasts for up to a few 100 ms after the stimulus is removed. Upon saccade onset all three visual input signals representing the pre-saccadic stimulus location are truncated.
- The synaptic connections from the visual population and saccade population to the combination population are dynamically adjusted through unsupervised competitive learning using a Hebbian synaptic learning rule. This forces neurons in the combination population to learn to represent different combinations of retinal stimulus location and saccade target location.
- The synapses from the combination population to the remapping population are dynamically adjusted through associative learning with a Hebbian synaptic learning rule. This layer of synapses effectively implements supervised pattern association learning from the combination population to the remapping population. Specifically, these synapses learn to map particular combinations of the pre-saccadic location of a visual target and target location of the impending saccade represented by the combination neurons onto the corresponding post-saccadic location of the visual target represented by the remapping neurons.
- The head remains stationary across saccades ensuring that the head-centered visual space remains stable during these brief periods.

### 2.2. Details of model components

The visual input population functions as an eye-centered map of the visual stimuli present within a scene. However, neurons in this population have a delayed update time of a few 100 ms in their response to a saccade, which alters the visual representation in eye-centered space. This response inertia is then propagated to the combination population. In constrast, the remapping neurons do not have such long delays in their responses to saccades. The effect of this is to allow an inter-temporal association to occur in the synaptic connections from the combination population, which represents the scene before the saccade, to the remapping population, which represents the scene after the saccade.

It is proposed that a population of visual neurons with long post-saccadic response delays during saccades of the order of hundreds of milliseconds could arise due to the accumulation of axonal conduction delays (Girard et al., 2001) and neuronal response delays of the order of tens of milliseconds as visual signals are propagated between a number of subcortical and cortical brain regions, including being propagated back and forth acoss recurrent connections within individual regions. Furthermore, the memory trace response of a visual neuron after a stimulus has been removed from its receptive field, either due to the extinction of the stimulus or a saccade, may be enhanced by biological factors such as long synaptic time constants that keep the postsynaptic neuron active for tens of milliseconds after the presynaptic signals have been extinguished (Evans and Stringer, 2012), and local recurrent circuits that may help to maintain loops of activity after the stimulus is removed (Elliffe et al., 2000). Such trace responses representing a pre-saccadic stimulus location may also contribute to the delayed response of the visual population to the new location of the stimulus after a saccade. It is proposed that a small subset of visual neurons, intermixed within various stages of processing, will have the required long post-saccadic response latencies. Consequently, the proposed population of visual neurons may be distributed across a number of linked brain areas, including visual areas V1 to V4 and MT, all of which project directly or indirectly to the areas within which remapping has been found (Blatt et al., 1990).

However, in the modeling study described below, it was decided to focus on the remapping dynamics, themselves, rather than attempt to simulate how the visual population develops long post-saccadic response latencies over a series of subcortical and cortical stages. Therefore, the post-saccadic response latencies are imposed directly on the visual population in the model simulations presented here.

The saccade population functions like an eye-centered motor encoding of impending saccades. Critically, this population represents the eye-centered target location of a saccade, both in advance of the saccade and for a few 100 ms after the saccade has been executed. Many examples of neurons encoding impending saccades have been identified, for example, FEF movement cells (Bruce and Goldberg, 1985), LIP neurons (Ipata et al., 2006), and SC neurons (Wurtz and Goldberg, 1972). Neurons in FEF have also been identified with explicit post-saccadic responses (Bizzi, 1968; Bruce and Goldberg, 1985).

The remapping population also functions like an eye-centered map of the stimuli present within the visual scene, but which is endowed with some extra characteristics to imitate the neuronal responses observed in LIP. These extra response characteristics include a specific response latency to a stimulus that is flashed in the neuron's receptive field, a trace response to a recently extinguished stimulus, and the truncation of this trace response as a result of a saccade onset (Duhamel et al., 1992). The neurons in this population have different response latencies that result in different remapping latencies. The neuronal trace response and the truncation of the trace response as a result of saccade onset were both included to replicate the findings of Kusunoki and Goldberg (2003).

The remapping population also receives associatively modifiable synaptic connections from the combination population, which represents a combination of the time-delayed visual stimulus location and saccade target location. The time-delayed responses of the input populations, leading to a corresponding delay in the representation carried by the combination population, leads to a time difference of a few 100 ms between the representations in the combination population and remapping population. This allows the network to learn associations between particular combinations of pre-saccadic stimulus location and saccade target location represented by the combination population and the resulting post-saccadic stimulus location represented by the remapping population. Thus, the goal was for the model to self-organize its synaptic connections by visually-guided learning in order that the remapping population is able to predict the post-saccadic stimulus location, as well as replicate various other experimentally observed remapping dynamics.

The combination population received associatively modifiable synaptic connections from the visual population and saccade population. Neurons in the combination population learned to encode unique combinations of the impending saccade vector and the present pre-saccadic retinal location of the visual stimulus. Neurons in this population had their firing rate threshold set so that they would only respond if they received simultaneous input from both the visual population and saccade population. The combination population could potentially exist in the same cortical region as the remapping population itself.

It is assumed that the head and visual stimuli often remain stationary during the time course of a saccade, and this is a reasonable assumption given the known statistics of how primates move their head and eyes. Specifically, a primate adjusts its gaze more frequently by moving its eyes rather than its head (Freedman and Sparks, 1997). Evidence for this during exploration of natural environments with free eye, head and body movements has been reported by Einhäuser et al. (2007).

### 2.3. Self-organization process

The self-organization of the synaptic connections in the model happens as follows. During a training trial, the stimulus remains stationary in head-centered space while a single saccade is performed. Before the planned saccade is initiated, both the visual population and saccade population will be active, encoding the location of a visual stimulus in the scene and the target location of the impending saccade, respectively. This will stimulate a subset of neurons in the combination population, and through competitive learning these neurons will become tuned to this particular combination of pre-saccadic stimulus location and saccade target location. During the course of training over many saccades, the model should develop a large number of differently tuned combination neurons, which encode all combinations of stimulus location and saccade target location that occured during training.

When each saccade is initiated, the visual population and saccade population will have delayed responses to the saccade of a few 100 ms. This allows the corresponding set of combination neurons to continue to fire for this period. However, the responses of the remapping population are not subject to the same time delays as the visual population and saccade population. Therefore, immediately after the saccade, the remapping population will reflect the post-saccadic location of the stimulus. In this situation, the active combination neurons representing the pre-saccadic activity will be associated onto the remapping neurons that reflect the post-saccadic stimulus location.

A few 100 ms after the saccade, the visual population will begin to represent the post-saccadic location of the stimulus. However, the saccade population will cease responding. This will cause the combination population to cease responding as well due to the firing rate threshold. Thus, further associative learning in the connections from the combination population to the remapping population will cease at this point for the current saccade.

After training, a particular combination of pre-saccadic stimulus location and saccade target location represented in the visual population and saccade population will stimulate the corresponding subset of combination neurons. These combination neurons, in turn, will stimulate the remapping neurons that represent the corresponding post-saccadic stimulus location.

### 2.4. Predicted model behavior

The remapping activity in the model is mediated by the combination population. Successful self-organization of the synaptic connections from the visual population and saccade population to the combination population produces neurons in the combination population that respond to unique combinations of the pre-saccadic retinal stimulus location and saccade target location encoded in the corresponding input populations. Simultaneously, successful self-organization of the synaptic connections from the combination population to remapping population allows subsequent activity from the combination population to stimulate neurons in the remapping population that represent the post-saccadic retinal stimulus location corresponding to the current combination of pre-saccadic stimulus location and saccade target location. It was hypothesized that the remapping population within a properly self-organized model should be able to replicate the experimental observations described above in the following ways:

- *Predictive remapping* should happen when activity in the visual population representing the pre-saccadic stimulus location and activity in the saccade population representing the saccade target location stimulate the corresponding combination neurons, which then stimulate the remapping neurons that represent the corresponding post-saccadic stimulus location.
- *Pre-saccadic remapping*, whereby remapping begins before saccade onset, should occur by the same means as predictive remapping described above. Although not simulated here, the broad distribution of response latencies in both predictive and pre-saccadic remapping could naturally emerge as a result of variability in axonal delays or neuronal and synaptic time constants.
- *Trace remapping* would occur in the remapping population because the visual input population would continue to represent a stimulus a few 100 ms after the stimulus offset. This would allow memory activity within the visual population reflecting a recently extinguished stimulus, combined with activity from the saccade population representing the intended saccade target location, to stimulate remapping neurons that reflect what would have been the corresponding post-saccadic stimulus location.
- *Responsiveness shift* in the remapping population around the time of a saccade may also be accounted for with this model. First, consider a stimulus flashed in the spatial location where the receptive field is located before the saccade. When the stimulus is flashed at increasing times before saccade onset, the trace response truncation in the remapping neurons causes a declining response as measured after stimulus onset. When the stimulus is flashed after saccade onset, there is no response. Second, consider a stimulus flashed in the spatial location where the receptive field is located after the saccade. When the stimulus is flashed at increasing times before saccade onset, remapping causes an increasing response in the remapping population as measured after stimulus onset. When the stimulus is flashed after saccade onset, there is a purely visual response in the remapping population.
- *Spatially independent remapping* would occur as long as the model was extensively trained on many different combinations of pre-saccadic stimulus location and saccade target location. After training, this would enable individual neurons in the remapping population to remap activity from multiple different spatial locations and directions.

### 2.5. Network model architecture

The network model consisted of four populations of neurons, the names and interconnectivity of which is shown in Figure 1.

There were two input populations that represented corresponding one-dimensional retinotopic spaces. The first input population, the visual population, was purely visual and represented the retinal location of visual stimuli. While the second input population, the saccade population, encoded saccade plans by representing the retinal target locations of impending saccades. The visual population, the size of which was denoted by *N*^V^, consisted of visual neurons representing [−45°, 45°] of eye-centered visual space. The saccade population, the size of which was denoted by *N*^S^, consisted of saccade planning neurons representing [−30°, 30°] of eye-centered saccade space. Each population represented the corresponding space by having neurons with a preference for each integer degree location in the space. Hence *N*^V^ = 91 and *N*^S^ = 61. Neither population had any topographic organization. However, simulation results will be presented topographically according to neuron preferences in order to facilitate inspection of the model behavior.

The combination population consisted of *N*^C^ combination neurons that each received synaptic connections from a randomly assigned subpopulation of neurons from the visual and saccade populations. Specifically, each combination neuron received inputs from a subpopulation consisting of ϕ^V^ and ϕ^S^ percent of the visual and saccade populations, respectively. Hence the total number of afferents per combination neuron was (*N*^V^ϕ^V^ + *N*^S^ϕ^S^)/100.

The remapping population, the size of which was denoted by *N*^R^, also represented the retinotopic locations of visual stimuli. This population had the same encoding and structure as the visual input population. It consisted of visual neurons representing [−45°, 45°] of eye-centered visual space, where each integer position in the space was represented by a corresponding neuron with a preference for that location. Hence, the number of remapping neurons was *N*^R^ = 91. This map was also not topographically organized. However, simulation results will present the remapping population topographically in terms of neuronal preference in order to aid analysis of the model performance. Each remapping neuron received synaptic connections from a randomly assigned subpopulation of neurons from the combination population. Specifically, each remapping neuron received inputs from a subpopulation consisting of ϕ^C^ percent of the combination neurons. Hence the total number of afferents per remapping neuron was (*N*^C^ϕ^C^)/100.

All synaptic connections between neurons were initially set to a random weight in the interval [0, 1] and subsequently normalized as described in Section 2.9.

### 2.6. Stimuli

The relationship between the eye-centered location *r* of a visual stimulus, the head-centered eye position *e*, and the head-centered location *h* of the same visual stimulus is given by the Equation *h* = *e* + *r*. The space of head-centered visual stimulus locations, *h*, covered the interval [−45°, 45°]. This meant that visual targets could only be located within this given region of head-centered space. Visual targets were always stationary in head-centered space within a given trial, both in training and testing. The space of eye-centered visual stimulus locations represented by both the visual and remapping populations also covered [−45°, 45°]. All trials started with the eye fixating straight ahead at *e* = 0° in head-centered space. The space of retinotopic saccade target locations represented by the saccade population covered [−30°, 30°], which meant that the saccade population could only represent this range of saccades. Since all trials only had one saccade at most, this implied that the final head-centered eye position *e* was also confined within the given saccade plan space [−30°, 30°]. However, for any given head-centered stimulus location, the retinal saccade target location was in practice further bounded to ensure that the post-saccadic retinal stimulus location remained within [−45°, 45°]. The model was first trained, and then subsequently tested on a range of different tasks, described below, to characterize the various receptive field properties of all neurons in the model. All saccades are performed at a constant velocity of 300°/*s*.

### 2.7. Training the network

A training epoch consisted of *M* different trials, each of which involved the model performing a saccade while a visual stimulus was present at some location in the head-centered visual space. The head-centered stimulus location and saccade was varied randomly between trials. Each trial thus trained the network on a particular remapping.

In more detail, every trial *i* began with fixation straight ahead, with initial head-centered eye position *e*^0^_*i*_ = 0. For each trial, a stimulus was placed in a head-centered location *h*_*i*_ for the duration of the trial. This corresponded to an initial retinotopic stimulus location *r*^0^_*i*_ = *h*_*i*_ − *e*^0^_*i*_ = *h*_*i*_. Then the model performed a saccade to a retinal target location *s*_*i*_ that brought the stimulus onto a final retinal location *r*_*i*_ = *r*^0^_*i*_ − *s*_*i*_ after the saccade. For each trial, the head-centered stimulus location *h*_*i*_ and eye-centered saccade *s*_*i*_ were picked randomly, but with the constraint that the resulting saccade was no less than 10° in magnitude, and that *r*_*i*_ remained within the retinal space.

**Figure 2:**
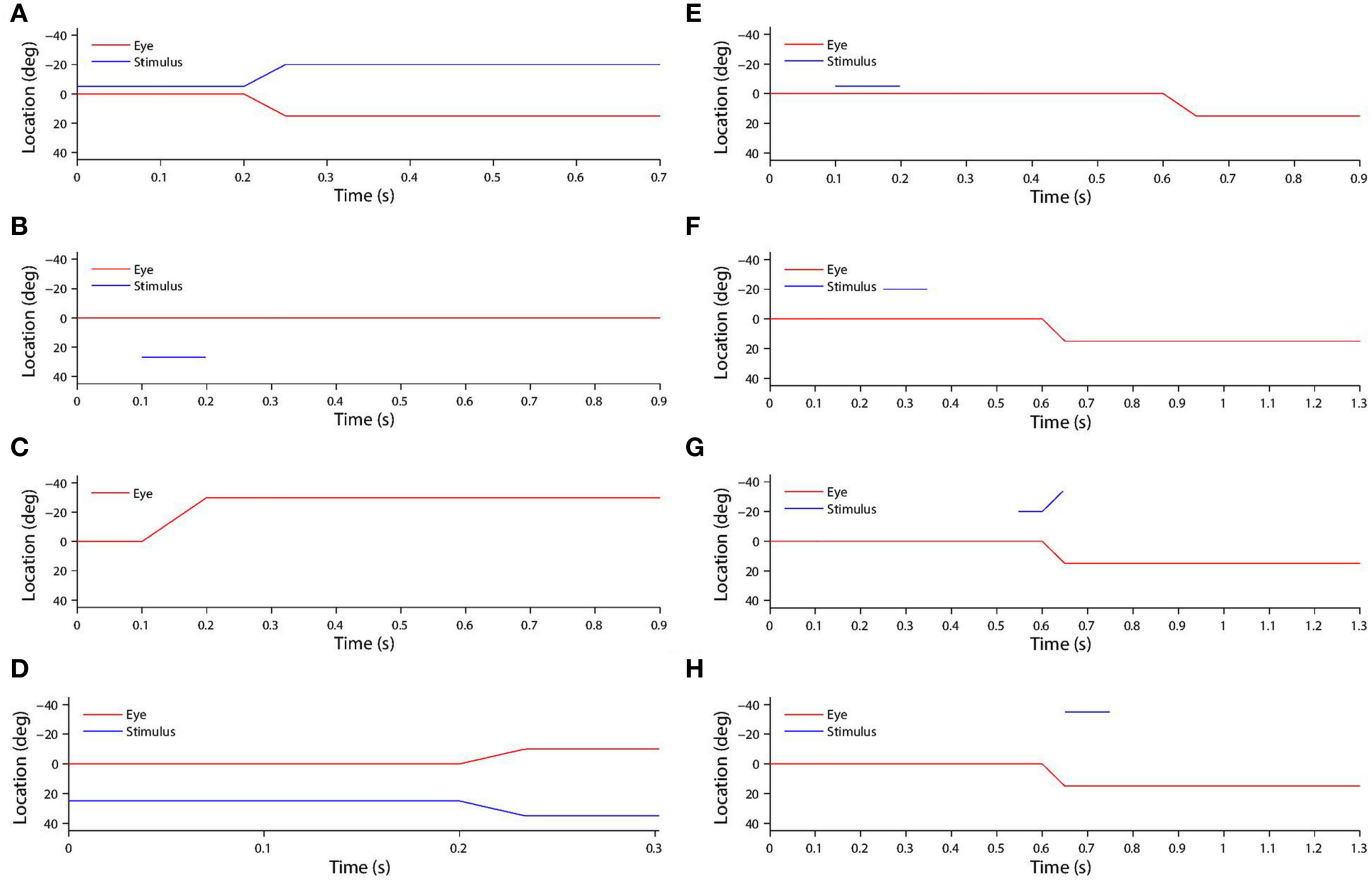
**(A)** Shows a typical training trial. The head centered eye position (red) and eye-centered visual stimulus location (blue) are plotted against time. The trial began while fixation was straight ahead (eye position was at 0°) and the stimulus was located at −5°. A saccade of 15° was initiated at 200 ms with a constant velocity of 300°/*s*, and was completed in 50 ms. After the saccade, another 450 ms fixation period at the new eye position 15° was performed, while the stimulus had moved to −5°-15° = −20° on the retina. **(B)** shows a typical stimulus control task trial. The task began while fixation was straight ahead. The stimulus was presented at retinal location 32° during the period 100–200 ms. After stimulus offset, there was a 700 ms period of maintained fixation. **(C)** shows a typical saccade control task trial. The task began while fixation was straight ahead. A saccade of −30° was initiated at 100 ms, and with a constant velocity of −300°/*s*. It was completed 100 ms later. After saccade completion, fixation was maintained at the new eye position −30° for another 700 ms. **(D)** show a typical probe task trial. The trial began while fixation was straight ahead and a stimulus was presented at 25°. A saccade of −10° began at 200 ms, and with a constant velocity of −300°/*s*. It was completed 100/3 ms later. **(E)** shows a typical single step task trial. The trial began while fixation was straight ahead. At 100 ms a stimulus was presented at −5° for 100 ms. Then at 600 ms a 15° saccade was initiated. The constant saccade velocity of 300°/*s* meant the saccade ended after 50 ms. After saccade completion, the fixation was maintained for another 250 ms at the new eye position of 15°. **(F,G,H)** show three typical delayed stimulus flash task trials with the given stimulus onset times of 250 ms **(F)**, 550 ms **(G)** and 650 ms **(H)**. All plots are current receptive field trials for a neuron with retinal receptive field −20°. In all three cases the stimulus is located in head-centered location −20°. The saccade is 15° in all three cases, and is initiated at 600 ms and lasts for 50 ms.

The saccade was initiated after 200 ms of initial fixation. Then a post-saccadic fixation period of 300 ms combined with some time for the saccade itself brought the total trial length to approximately 700 ms. Figure 2A shows the typical time course of a training trial.

### 2.8. Testing the network

#### 2.8.1. Stimulus control task

In stimulus control tasks, a visual target was briefly flashed while fixation was maintained throughout the trial. A given trial presented the stimulus in a unique retinal location. Since there was one such trial for each integer location in the retinal space [−45°, 45°] there were 91 trials in total. For each remapping neuron, two particular trials were of interest. Firstly, the trial corresponding to the retinal preference of the given neuron was used to assess the visual response latency of the given neuron. This was later compared to the latency of the neuron when testing remapping, so as to reveal potential predictive remapping. Secondly, the trial corresponding to the retinal location in which, during training, the stimulus had been presented pre-saccadically before remapping into the receptive field of the given neuron. This trial was necessary in order to confirm that the response of the neuron, when testing for remapping, was not simply due to the presence of the stimulus in the visual field. This control was also used by Heiser et al. (2005), Heiser and Colby (2006), Berman et al. (2007), and Dunn et al. (2010). Figure 2B shows the time course of a typical stimulus control task trial.

#### 2.8.2. Saccade control task

In saccade control tasks, a single saccade was executed while no stimulus was present in the visual field. A given trial required performing a unique saccade. Since there was one such trial for each integer location in the space of retinotopic saccade target locations [−30°, 30°], there were 61 trials in total. For each remapping neuron, the task corresponded to the saccade performed during training that set up remapping into the receptive field of the given neuron. This was necessary to confirm that the response of the neuron, when testing for remapping, was not simply due to the saccade execution. This control was also used by Heiser et al. (2005), Heiser and Colby (2006), Berman et al. (2007), and Dunn et al. (2010). Figure 2C shows the time course of a typical saccade control task trial.

#### 2.8.3. Probe task

In probe tasks, a single saccade was executed while a stimulus was presented for the full duration of the trial. A given trial involved a unique combination of stimulus location and saccade target location. Since there was one such trial for each combination of integer positions in each of the two spaces, the total number of trials was 61 × 91 = 5551. For each combination neuron, this full set of trials allowed the decoding of what combination of stimulus location and saccade target location the neuron was responsive to. This information was further used to analyze the functional connectivity of the weight vectors of neurons in both the combination and remapping populations. Figure 2D shows the time course of a typical probe task trial.

#### 2.8.4. Single step task

In single step tasks, a stimulus was briefly flashed and a saccade was subsequently performed. A given single step task trial corresponded to a specific training trial in the sense that it involved the same combination of stimulus location and saccade target location. The purpose of this task was to measure the remapping that the corresponding training trial was meant to have taught the network. The difference between the single step task trials and the training trials was that in single step trials the stimulus was flashed for only a short period, as in Duhamel et al. (1992), Heiser et al. (2005), Heiser and Colby (2006), Berman et al. (2007), and Dunn et al. (2010), while in training trials it remained visible for the full duration of the trial. Figure 2E shows the time course of a typical single step task trial.

#### 2.8.5. Delayed stimulus flash task

The *i*th training trial involved a saccade *s*_*i*_ that brought the receptive field of a remapping neuron to the head-centered location of the visual stimulus. For each training trial, there were two families of delayed stimulus flash tasks conducted during testing. In each delayed stimulus flash task, a stimulus was briefly flashed at some time with respect to the same saccade *s*_*i*_ that was performed during the *i*th training trial. In one family of task trials, the stimulus was flashed in the head-centered location corresponding to where the receptive field of the remapping neuron was located in the training trial before the saccade. Each of these trials was referred to as a *current receptive field trial*. In the other family of task trials, the stimulus was flashed in the head-centered location corresponding to where the receptive field of the remapping neuron was located in the training trial after the saccade. Each of these trials was referred to as a *future receptive field trial*. Both families had the same number of trials, each of which corresponded to a given stimulus onset time ranging from 100 ms to 700 ms in increments of 50 ms. This resulted in 13 trials in each family. The purpose of this task was to measure the time course of the responsiveness of the neuron to a stimulus presented in the current and future receptive fields around the time of a saccade, exactly as done by Nakamura and Colby (2002) and Kusunoki and Goldberg (2003). Figures 2F–H show the time course of a typical delayed stimulus flash task trial.

### 2.9. Neuronal and synaptic dynamics

For all simulation experiments, there was at most one saccade performed during each training or testing trial, with the saccade performed from fixation straight ahead to retinal location *s*. Moreover, there was never more than a single visual stimulus present during a trial, which was kept fixed at a head-centered location *h*. For those trials with a stimulus present, there was only a single time of onset for the stimulus during the trial. The times of the saccade onset, visual stimulus onset and visual stimulus offset are denoted by *t*^SACC^, *t*^STIM^_ON_ and *t*^STIM^_OFF_, respectively. Let *e*(*t*) denote the head-centered eye position at time *t* during a trial, then Ψ(*t*) = *h* − *e*(*t*) is the retinal location of the visual stimulus at time *t*. However, Ψ(*t*) is set to ∞ if there is no visible visual stimulus present at time *t*. Likewise, if there is no saccade in the trial then *s* is set to ∞.

#### 2.9.1. Visual population

Each neuron 1 ≤ *i* ≤ *N*^V^ in the visual population was assigned a unique retinal preference α^V^_*i*_ ∈ [−45°, 45°]. The firing rate *v*^V^_*i*_(*t*) of visual neuron *i* was governed by

(1) $$\begin{array}{l} \tau_{v}^{\text{V}}\frac{dv_{i}^{\text{V}}}{dt}=\delta (t-t_{\text{ON}}^{\text{STIM}})\exp\left(-\frac{{\left(\alpha_{i}^{\text{V}}-\Psi (t)\right)}^{2}}{2\sigma_{\text{V}}^{2}}\right)\\ \;-\;\delta (t-t^{\text{SACC}}+\Delta^{V})v_{i}^{\text{V}}\\ \;+\;\delta (t-t^{\text{SACC}}+\Delta^{V})\exp\left(-\frac{{\left(\alpha_{i}^{\text{V}}-\Psi (t)\right)}^{2}}{2\sigma_{\text{V}}^{2}}\right) \end{array}$$

The time constant τ^V^_*v*_ was uniform for all neurons in the visual input population. The terms on the right hand side of Equation (1) were as follows.

The first term on the right hand side of Equation (1) caused an instantaneous rise in the neuronal firing rate due to the onset of a stimulus. The rise occurred at the time of the stimulus onset, and the magnitude of the rise was a Gaussian function of the distance between the neuron's preferred retinal location α^V^_*i*_ and the location of the stimulus Ψ(*t*). Hence the magnitude of the rise fell off radially as the stimulus location shifted away from the receptive field center. The standard deviation of the Gaussian tuning curve was uniform across all neurons, and was given by σ_V_.

The visual neurons are assumed to maintain their activity, even when the stimulus is removed from the pre-saccadic retinal location, up to a period of Δ^*V*^ past the saccade onset. This may be effected in the brain by the presence of, for example, local recurrent loops within and between layers leading to attractor states. However, we do not model these local recurrent networks explicitly here. Instead, to achieve this effect there is no exponential decay term included on the right hand side of Equation (1).

The second term on the right hand side of Equation (1) caused an instantaneous decline in the neuron's firing rate due to the initiation of a saccade, as in Duhamel et al. (1992). The decline occured at a time Δ^*V*^ after the saccade onset. The magnitude of the decline was equal to the current firing rate in order to eliminate the activity of the neuron due to previous stimulation by the pre-saccadic location of the target.

The third term on the right hand side of Equation (1) caused an instantaneous rise in firing rate due to the the new retinal location of a visual stimulus after a saccade. The rise occurred at a time Δ^*V*^ after the saccade onset, and the magnitude of the rise was a Gaussian function of the distance between the neuron's preferred retinal location α^V^_*i*_ and the post-saccadic location of the stimulus Ψ(*t*). The firing rate dynamics of the visual neurons specified above were phenomenological and exogenous to the model.

#### 2.9.2. Saccade population

Each neuron 1 ≤ *i* ≤ *N*^S^ in the saccade population was assigned a unique saccade preference β^S^_*i*_ ∈ [−30°, 30°]. The firing rate *v*^S^_*i*_(*t*) of saccade neuron *i* was governed by

(2) $$\tau_{v}^{\text{S}}\frac{dv_{i}^{\text{S}}}{dt}=-v_{i}^{\text{S}}+I(t-t^{\text{SACC}},\Delta_{\text{PRE}}^{\text{S}},\Delta_{\text{POST}}^{\text{S}})\exp\left(-\frac{{\left(\beta_{i}^{\text{S}}-s\right)}^{2}}{2\sigma_{\text{S}}^{2}}\right)$$

where

(3) $$I(x,y,z)=\{\begin{array}{ll} 1 & \text{if }-y\leq x\leq z\\ 0 & \text{otherwise} \end{array}$$

The time constant τ^S^_*v*_ was uniform for all neurons in the saccade population. The indicator function *I* governed when the saccade neurons were activated with respect to the time of saccade onset *t*^SACC^. Specifically, *I* had a value of 1 whenever *t* was in the interval [*t*^SACC^ − Δ^S^_PRE_, *t*^SACC^ + Δ^S^_POST_], where Δ^S^_PRE_ and Δ^S^_POST_ were positive time delays representing how soon in advance and how long after saccade onset the saccade neurons started and stopped responding. During this time interval, the firing rate of the saccade neuron was driven up by a Gaussian function of the difference between the saccade preference of the neuron β^S^_*i*_ and the actual saccade plan *s*, with a standard deviation of σ_S_.

It was important that the saccade neurons remained active for some short period Δ^S^_POST_ after the saccade onset in order to keep the combination neurons active for a similar period after the saccade. This was necessary to enable the combination neurons to learn associations with the appropriate remapping neurons representing the post-saccadic retinal stimulus location.

#### 2.9.3. Combination population

The combination neurons were driven dynamically by synaptic inputs from presynaptic visual neurons and saccade neurons. Consequently, for each neuron 1 ≤ *i* ≤ *N*^C^ in the combination population there were two dynamical quantities defined: an internal activation *h*^C^_*i*_(*t*) and a firing rate *v*^C^_*i*_(*t*).

The activation *h*^C^_*i*_ of combination neuron *i* was governed by

(4) $$\begin{array}{l} \tau_{h}^{\text{C}}\frac{dh_{i}^{\text{C}}}{dt}=-h_{i}^{\text{C}}+\;\psi^{\text{V}\to \text{C}}\sum_{j\;=\;1}^{N^{\text{V}}}w_{ij}^{\text{V}\to \text{C}}v_{j}^{\text{V}}\\ \;+\;\psi^{\text{S}\to \text{C}}\sum_{j\;=\;1}^{N^{\text{S}}}w_{ij}^{\text{S}\to \text{C}}v_{j}^{\text{S}}-w_{\text{INHB}}^{\text{C}}\sum_{j\;=\;1}^{N^{\text{C}}}v_{j}^{\text{C}} \end{array}$$

The time constant τ^C^_*h*_ was uniform for all neurons in the combination population. The terms on the right hand side of Equation (4) were as follows. The first term represented the constant leak in the activation. The second term represented the excitatory synaptic input to the combination neuron from the visual population, where *w*^V → C^_*ij*_ was the weight of the synapse from visual neuron *j* to combination neuron *i*. The third term represented the excitatory synaptic input to the combination neuron from the saccade population, where *w*^S → C^_*ij*_ was the weight of the synapse from saccade neuron *j* to combination neuron *i*. The fourth term represents inhibitory feedback from the population of combination neurons, where *w*^C^_INHB_ is a constant scaling parameter. This implements competition between combination neurons and helps to keep the overall level of activity within the combination population constant, which is required to facilitate the competitive learning process in this population.

The firing rate *v*^C^_*i*_ of combination neuron *i* was a function of the activation and was given by the sigmoid function

(5) $$v_{i}^{\text{C}}=\frac{1}{1+\exp\left(-2\varphi^{\text{C}}(h_{i}^{\text{C}}-\theta^{\text{C}})\right)}$$

where φ^C^ was the sigmoid slope, and θ^C^ was the threshold.

Since the combination neurons were driven by inputs from the visual population and saccade population, the combination neurons inherited the response delays from these two input populations. This ensured that the combination neurons representing a particular combination of pre-saccadic retinal stimulus location and saccade target location stayed active for a brief period after the saccade. This in turn allowed the combination neurons to learn associations with remapping neurons representing the corrresponding post-saccadic retinal stimulus location.

During training, each synaptic weight *w*^V → C^_*ij*_ was modified according to a Hebbian learning rule

(6) $$\frac{dw_{ij}^{\text{V}\to \text{C}}}{dt}=\varrho^{\text{V}\to \text{C}}v_{i}^{\text{C}}v_{j}^{\text{V}}$$

where ϱ^V → C^ was the learning rate. Similarly, each synaptic weight *w*^S → C^_*ij*_ was modified according to a Hebbian learning rule

(7) $$\frac{dw_{ij}^{\text{S}\to \text{C}}}{dt}=\varrho^{\text{S}\to \text{C}}v_{i}^{\text{C}}v_{j}^{\text{S}}$$

where ϱ^S → C^ was the learning rate.

Unbounded growth of the synaptic weights during training was prevented by continually renormalizing the synaptic weight vectors to ensure

(8) $$\sum_{j}{\left(w_{ij}^{\text{V}\to \text{C}}\right)}^{2}=1$$

and

(9) $$\sum_{j}{\left(w_{ij}^{\text{S}\to \text{C}}\right)}^{2}=1$$

for each post-synaptic combination neuron *i* after each weight update (Dayan and Abbott, 2001). Experimental evidence for renormalization of synaptic weights in the brain has been provided by Royer and Paré (2003).

#### 2.9.4. Remapping population

Each neuron 1 ≤ *i* ≤ *N*^R^ in the remapping population was assigned a unique retinal preference α^R^_*i*_ ∈ [−45°, 45°]. The remapping neurons were driven dynamically by synaptic inputs from presynaptic combination neurons in addition to visual inputs. Consequently, for each remapping neuron *i* there were two dynamical quantities defined: an internal activation *h*^R^_*i*_(*t*) and a firing rate *v*^R^_*i*_(*t*).

The activation *h*^R^_*i*_ of remapping neuron *i* was governed by

(10) $$\begin{array}{l} \tau_{h}^{\text{R}}\frac{dh_{i}^{\text{R}}}{dt}=-h_{i}^{\text{R}}+\psi^{\text{C}\to \text{R}}\sum_{j\;=\;1}^{N^{\text{C}}}w_{ij}^{\text{C}\to \text{R}}v_{j}^{\text{C}}\\ \;-\;w_{\text{INHB}}^{\text{R}}\sum_{j\;=\;1}^{N^{\text{R}}}v_{j}^{\text{R}}+\;K_{i} \end{array}$$

The time constant τ^R^_*h*_ was uniform for all neurons in the remapping population. The terms on the right hand side of Equation (10) were as follows. The first term represented the constant leak in the activation. The second term represented the excitatory synaptic input to the remapping neuron from the combination population, where *w*^C → R^_*ij*_ was the weight of the synapse from combination neuron *j* to the remapping neuron *i*. The third term represents inhibitory feedback from the population of remapping neurons, where *w*^R^_INHB_ is a constant scaling parameter. The fourth term *K*_*i*_ represented the external visual input to remapping neuron *i*. This was governed by

(11) $$\begin{array}{l} \tau^{\text{K}}\frac{dK_{i}}{dt}=-K_{i}+\psi^{\text{K}}I\left(t-t_{\text{ON}}^{\text{STIM}},-\Gamma_{i}^{\text{K}},t_{\text{OFF}}^{\text{STIM}}-t_{\text{ON}}^{\text{STIM}}\right)\\ \;\exp\left(-\frac{{\left(\alpha_{i}^{\text{R}}-\Psi (t)\right)}^{2}}{2\sigma_{\text{R}}^{2}}\right)\\ \;-\;\delta (t-t^{\text{SACC}}-\Delta^{K})K_{i}+P_{i} \end{array}$$

The time constant τ^K^ was uniform for all neurons in the remapping population, and was relatively short in all experiments, e.g., 20 ms. The terms on the right hand side of Equation (11) were as follows.

The first term on the right hand side of Equation (11) represented a constant leak.

The second term on the right hand side of Equation (11) represented the visual drive due to the presence of a visual stimulus in the visual receptive field of the neuron. The indicator function *I*, defined by Equation (3), governed when the remapping neurons were activated with respect to the times of stimulus onset and stimulus offset. Specifically, *I* had a value of 1 whenever *t* was in the interval [*t*^STIM^_ON_ + Γ^K^_*i*_, *t*^STIM^_OFF_], where Γ^K^_*i*_ represented a positive onset delay for remapping neuron *i*. The values of Γ^K^_*i*_ were randomly drawn from 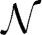
(0*ms*, 50*ms*), with negative values flipped to positive and all values above 80 ms clipped to this limit. During this time interval, *K*_*i*_ was driven up by a Gaussian function of the distance between the remapping neuron's preferred retinal location α^R^_*i*_ and the location of the stimulus Ψ(*t*), with a standard deviation of σ_R_.

The third term on the right hand side of Equation (11) implemented an instantaneous truncation due to the initiation of a saccade, which was effected a short time Δ^*K*^ after the saccade onset, as in Duhamel et al. (1992). Such saccade aligned activity truncation has also been implemented in other models such as those of Quaia et al. (1998) and Xing and Andersen (2000). The magnitude of the decline in *K*_*i*_ was equal to the present value of *K*_*i*_ in order to reduce this variable to a baseline value of zero.

The fourth term, *P*_*i*_, on the right hand side of Equation (11) was a tonic driving input specific to each remapping neuron *i*, and its dynamics were governed by

(12) $$\tau^{\text{P}}\frac{dP_{i}}{dt}\;=-P_{i}+\delta (t-t_{\text{OFF}}^{\text{STIM}})K_{i}-\delta (t-t^{\text{SACC}}-\Delta^{K})P_{i}$$

The time constant τ^P^ was relatively long in all experiments, e.g., 300 ms. *P*_*i*_ provided the driving input to *K*_*i*_ upon stimulus offset, giving the *i*^th^ remapping neuron a slow prolonged trace response when the stimulus was removed as in Duhamel et al. (1992). The terms on the right hand side of Equation (12) were as follows. The first term represented a constant leak. The second term caused an instantaneous rise in *P*_*i*_ upon stimulus offset. The magnitude of this rise was equal to *K*_*i*_. This resulted in *K*_*i*_, which was driven by *P*_*i*_, having a long trace response to a visual stimulus after its removal. The third term effected an instantaneous truncation of *P*_*i*_ a short time Δ^*K*^ after the saccade onset, as for *K*_*i*_.

The dynamics of the driving input signals *K*_*i*_ and *P*_*i*_ described by Equations (11) and (12) were purely phenomenological, designed through a process of experimentation to produce neuronal responses in the remapping population that matched the observed neuronal responses in LIP.

The firing rate *v*^R^_*i*_ of remapping neuron *i* was a function of the activation and was given by the sigmoid function

(13) $$v_{i}^{\text{R}}=\frac{1}{1+\exp\left(-2\varphi^{\text{R}}(h_{i}^{\text{R}}-\theta^{\text{R}})\right)}$$

where φ^R^ was the sigmoid slope, and θ^R^ was the threshold.

During training, each synaptic weight *w*^C → R^_*ij*_ was modified according to a Hebbian learning rule

(14) $$\frac{dw_{ij}^{\text{C}\to \text{R}}}{dt}=\varrho^{\text{C}\to \text{R}}v_{i}^{\text{R}}v_{j}^{\text{C}}$$

where ϱ^C → R^ was the learning rate. Unbounded growth of the synaptic weights during training was prevented by imposing the constraint

(15) $$\sum_{j}{\left(w_{ij}^{\text{C}\to \text{R}}\right)}^{2}=1$$

for each postsynaptic remapping neuron *i* after each weight update (Dayan and Abbott, 2001).

### 2.10. Numerical simulation

The system of differential equations were integrated numerically using the Forward-Euler scheme, where the numerical time step was set to one tenth of the smallest neuronal time constant among τ^V^_*v*_, τ^S^_*v*_, τ^C^_*h*_, τ^R^_*h*_, τ^K^ and τ^P^. All input stimuli were dynamically simulated and sampled at the same frequency as the time step.

### 2.11. Analysis

#### 2.11.1. Neuronal period response

The *period response* of a neuron was an analog to the average spike count rate over some time interval as measured in single unit recording neurophysiology studies. Specifically, let *v*(*t*) be the firing rate of a neuron for *t* ∈ [0, *T*] during a task trial. During the time period [*t*_1_, *t*_2_], the period response of the neuron was defined as

(16) $$\bar{v}=\frac{1}{t_{2}-t_{1}}\int_{t_{1}}^{t_{2}}v(t)dt$$

This integral was numerically integrated using the trapezoidal method.

#### 2.11.2. Neuronal response latency

The *response latency* of a neuron, that is the earliest time during the trial at which point the neuron is considered to have a response in its discharge, was defined as the time of the start of the first 30 ms window where the slope of the response curve was consistently above a minimal threshold (0.002). If no such time was found, then the neuron was considered unresponsive in the given trial.

#### 2.11.3. Remapping index

The *remapping index* of a remapping neuron was a measure of the strength of the activity of the neuron which could be attributed to remapping. For each single step task trial *i*, in which activity should be remapped by a saccade *s*_*i*_ into the retinal receptive field location *r*_*i*_, a remapping index is computed by first computing a visual index and a saccade index. These two indices measure how much of the neuronal activity in the single step task trial can be attributed to remapping when controlling for either purely visual or purely saccadic activity, respectively, as in Heiser et al. (2005), Heiser and Colby (2006), Berman et al. (2007), and Dunn et al. (2010). For both indices, the remapping activity was defined as the period response in a 300 ms saccade onset aligned epoch in the single step task.

The visual index was defined as the remapping activity in the single step task trial minus the period response from a corresponding stimulus control task trial, in which a visual stimulus was briefly flashed while fixation was maintained throughout the trial in the absence of a saccade. In particular, the visual stimulus was flashed at the pre-saccadic retinal location of the stimulus in the training trial that set up remapping into the retinal receptive field location *r*_*i*_. The period response from the stimulus control trial was computed over the interval [600, 900] ms. A 50 ms onset delay was used to compensate for the variability of visual onset delay among remapping neurons (Duhamel et al., 1992).

The saccade index was defined as the remapping activity in the single step task trial minus the period response from a corresponding saccade control task trial, in which the saccade *s*_*i*_ was performed with no visual stimulus present. The saccade control task performed the saccade *s*_*i*_ of the training trial that set up remapping into receptive field location *r*_*i*_. The period response from the saccade control trial was computed over a 300 ms interval aligned on the saccade onset.

Both of these indices were confined to [−1, 1], and the remapping index was the norm of a vector of the two indices, confining it to [0, $\sqrt{2}$]. A value of 0 indicated that no remapping activity can be attributed beyond either purely visual or purely saccadic activity. While, conversely, a value of $\sqrt{2}$ indicated that there was a maximal remapping response and that nothing could be attributed to purely visual or purely saccadic activity.

#### 2.11.4. Probe task decoding

For the purpose of understanding the functional structure of synaptic connectivity between the visual and saccade populations and the combination population, and also the connectivity between the combination population and the remapping population, it was necessary to decode the combination of retinal stimulus location and saccade to which a combination neuron was selective. To do this, we analyzed the period response of a combination neuron to all of the probe task trials. For the *i*^th^ probe task trial, let *r*_*i*_ be the retinal stimulus location at the start of the trial and let *s*_*i*_ be the saccade. Also, let *v*_*i*_ be the period response of the given combination neuron over the 50 ms epoch aligned at saccade initiation time for the *i*^th^ probe task trial. The selectivity of the given neuron in each of the two input spaces, encoded by the visual population and saccade population, was decoded by performing a center-of-mass calculation in the given space across all task trials. Hence the retinal selectivity of a given combination neuron was decoded as

(17) $$\frac{\sum_{i}r_{i}{\bar{v}}_{i}}{\sum_{i}{\bar{v}}_{i}}$$

where the summation is carried out over all task trials *i*. Likewise, the saccade selectivity was decoded as

(18) $$\frac{\sum_{i}s_{i}{\bar{v}}_{i}}{\sum_{i}{\bar{v}}_{i}}$$

## 3. Results

### 3.1. Predictive remapping

**Figure 3:**
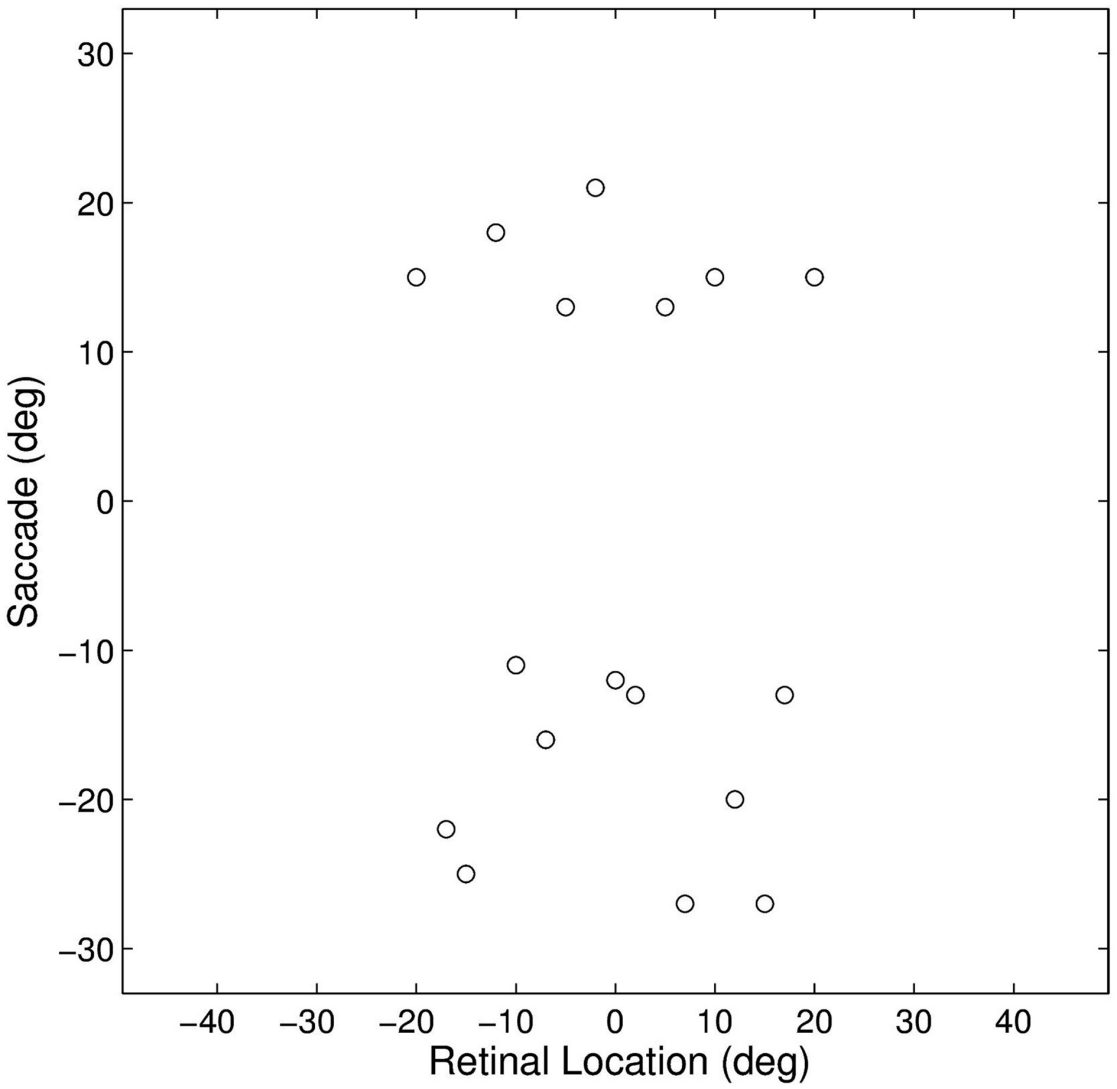
**Plot representing the inputs used for all of the single step task trials**. Each data point corresponds to the pre-saccadic retinal location of the flashed stimuli (abscissa) and the saccade performed (ordinate) in one of the trials. The 20° central portion of the saccade space, for which there are no trials, reflects the fact that all saccades were at least 10° in magnitude.

This experiment established how the model could develop the remapping dynamics described in Section 1. The model was trained and tested on a set of 17 different single step task trials. For each trial, a stimulus was initially flashed in a particular pre-saccadic retinal location and then a saccade was subsequently performed to a specific target location. These single step task trials are shown in Figure 3. The parameter values of the model are given in Table 1. It was hypothesized that each single step task trial would enable the remapping population to learn to remap visual activity from the pre-saccadic retinal location of the visual stimulus to the post-saccadic retinal location corresponding to the given saccade. Therefore, for each of the 17 different single step task trials, we analyzed the performance of the remapping neuron which had the same retinal preference α^R^_*i*_ as the post-saccadic stimulus location for that trial.

**Table 1:** **Parameters of the model**.

**Parameter**	**Symbol**	**Value**
**STIMULI**		
Number of remappings in training & testing	*M*	17
**VISUAL INPUT POPULATION**		
Population size	*N*^V^	91
Firing rate time constant	τ^V^_*v*_	1 s
Visual receptive field size	σ_V_	3°
Saccade supression delay	Δ^V^	280 ms
Learning rate: visual to combination neurons	ϱ^V → C^	0.1
**SACCADE INPUT POPULATION**		
Population size	*N*^S^	61
Firing rate time constant	τ^S^_*v*_	20 ms
Saccade receptive field size	σ_S_	3°
Pre-saccadic onset time	Δ^S^_PRE_	70 ms
Post-saccadic offset delay	Δ^S^_POST_	300 ms
Learning rate: saccade to combination neurons	ϱ^S → C^	0.1
**COMBINATION POPULATION**		
Population size	*N*^C^	1000
Activation time constant	τ^C^_*h*_	20 ms
Visual input coefficient	ψ^V → C^	10
Saccade input coefficient	ψ^S → C^	8
Lateral competition coefficient	*w*^C^_INHB_	0.1
Activation function slope	φ^C^	100
Activation function threshold	θ^C^	15
Learning rate: combination to remapping neurons	ϱ^C → R^	0.1
Visual input connectivity rate	ϕ^V^	5%
Saccade input connectivity rate	ϕ^S^	20%
**REMAPPING POPULATION**		
Population size	*N*^R^	5000
Activation time constant	τ^R^_*h*_	20 ms
Visual receptive field size	σ_R_	3°
Combination population input coefficient	ψ^C → R^	3
Lateal competition coefficient	*w*^R^_INHB_	0.6
Activation function slope	φ^R^	0.5
Activation function threshold	θ^R^	3
Combination population connectivity	ϕ^C^	100%
*K* time constant	τ^K^	20 ms
*K* coefficient	ψ^K^	8
*K* truncation delay	Δ^K^	0 ms
*P* time constant	τ^P^	300 ms

**Figure 4:**
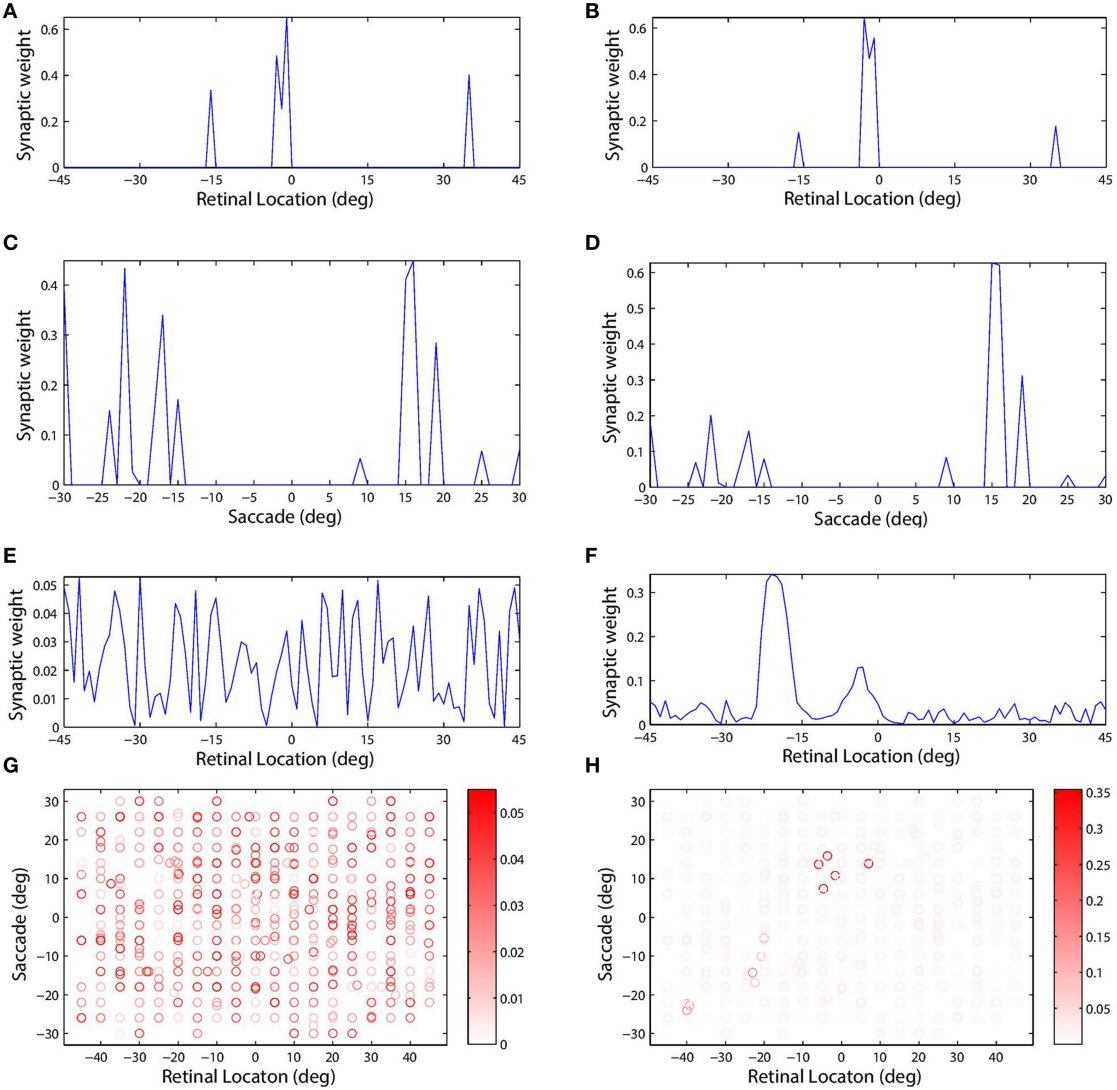
**The plots show synaptic connectivity in the self-organizing network model before training (left column) and after training (right column)**. The first **(A,B)** and second rows **(C,D)** show the weights of synapses afferent to combination neuron #181 from the visual and saccade input populations, respectively. These plots show synaptic weight as a function of the preferred location of the corresponding presynaptic input neuron in its input space. The third row **(E,F)** shows the weight of all efferent synaptic connections from the same combination neuron onto the remapping population. These plots show synaptic weight as a function of the preferred location of the corresponding postsynaptic remapping neuron in the retinal space. The scatterplots in the fourth row **(G,H)** show the decoded response preferences of all of the combination neurons in terms of stimulus retinal location (abscissa) and saccade target location (ordinate). These preferences were computed using the probe task decoding procedure described in Section 2.11. The boldness of each data point indicates the strength of the connection from that particular combination neuron to the remapping neuron with retinal preference −20°. This particular remapping neuron was indeed among those which combination neuron #181 was most strongly connected with after training **(F)**.

**Figure 5:**
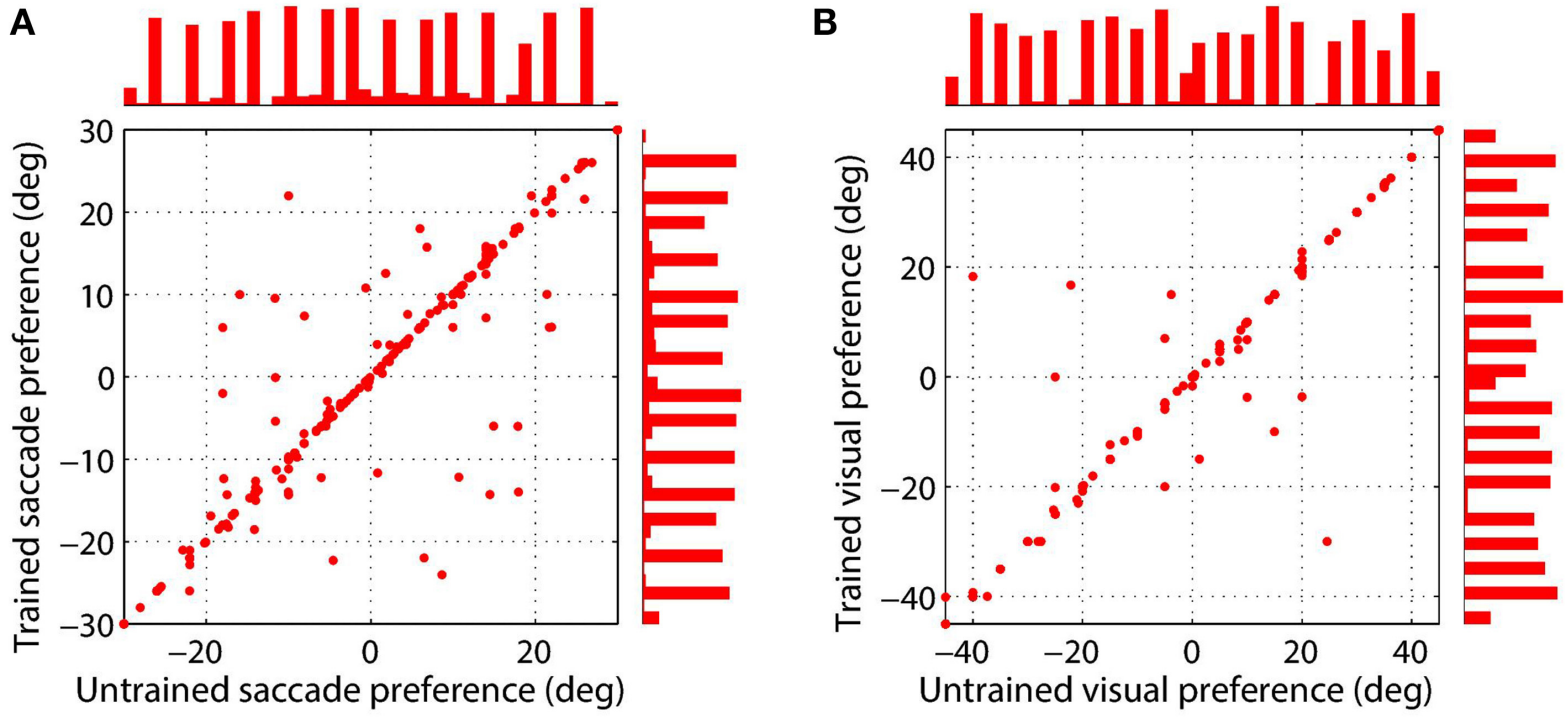
**Comparison of saccade target location (A) and stimulus location (B) preferences of combination neurons before training and after training**. Results are presented for those combination neurons for which both a saccade and stimulus location preference could be decoded. The left scatterplot shows the saccade preferences of combination neurons before and after training, where each data point corresponds to a different neuron. The right scatterplot presents the preferred stimulus location for each combination neuron before and after training. It can be seen that the data points in both plots are mostly clustered along the unit diagonal, which indicates that the saccade and stimulus location preferences are similar before and after training.

Figure 4 shows the synaptic connectivity in the model before and after training. The synaptic weights to combination neuron #181 from the visual and saccade populations before training are shown in (Figures 4A,C), respectively. The same weights after training are shown in (Figures 4B,D), respectively. After training, the combination neuron was clearly tuned to visual stimulus location −5° and saccade target location 15°. However, the effect of the training seemed to be that weaker afferents from other locations in the two spaces were diminished. This meant that the preference of a combination neuron was not greatly altered by training, but rather was largely governed by the initial random diluted connectivity. In other words, training tended to enhance the preexisting preferences. This finding is further supported by Figure 5, which compares the saccade and retinal location preferences of combination neurons before training and after training. Indeed, the correlation of saccade preferences of combination neurons before and after training was 0.975 (Figure 5A), while the correlation of retinal preferences before and after training was 0.990 (Figure 5B).

However, self-organization had a material impact on the structure of the efferent connections from a given neuron in the combination population to the remapping population. Combination neuron #181 projected to all neurons in the remapping population, i.e., there was full connectivity, and prior to training there was no structure in these efferents (Figure 4E). However, after training there was a clear topographic structure in which connections were potentiated to remapping neurons with a retinal preference close to either −5° or −20° (Figure 4F). The presence of strong connections to both of these retinal locations in the remapping population could be explained by the time course of the training trials and the preferences of this combination neuron. During training, the given combination neuron would have responded to its preferred combination of pre-saccadic stimulus retinal location and saccade. Moreover, the combination neuron would have started to respond in advance of the saccade due to the pre-saccadic response of the saccade population. Consequently, the efferent connections from the given combination neuron to remapping neurons representing the pre-saccadic stimulus location −5° would be strengthened by associative learning during the period of the pre-saccadic response of the saccade neurons, which was Δ^S^_PRE_ = 70 ms in this experiment. After the completion of the saccade, the stimulus is relocated to a new post-saccadic retinal location, which corresponds to the second peak of synaptic strength shown in Figure 4F, that is −20°. Meanwhile, the post-saccadic latency Δ^V^ = 280 ms in the visual population and the post-saccadic latency Δ^S^_POST_ = 300 ms in the saccade population will keep the given combination neuron active while both of these inputs remain simultaneously active after the saccade. Consequently, the efferent connections from this combination neuron to remapping neurons representing the post-saccadic stimulus location −20° will be potentiated through associative learning up to Δ^V^ = 280 ms after saccade onset. Notice that this relatively long duration of post-saccadic associative learning, relative to the period of pre-saccadic associative learning, explains why significantly more synaptic weight is devoted to remapping neurons representing the post-saccadic location.

Examining the weight vector of a remapping neuron with retinal preference −20° shows that before training it receives connections from combination neurons with a wide range of preferences (Figure 4G). However, after training, the presynaptic combination neurons with strong connections to the given remapping neuron all have very similar preferences to combination neuron #181 (Figure 4H). Also notice that the given remapping neuron does not receive connections from a full unit diagonal in the combined space of saccade and retinal location, just a localized region. This was because this model was only trained on a small subset of possible configurations.

**Figure 6:**
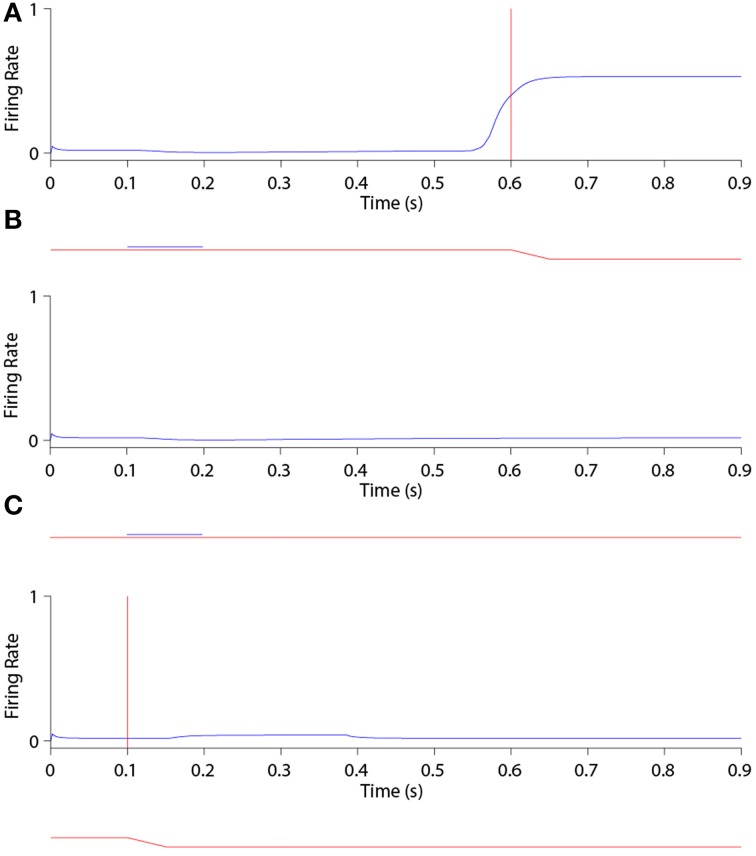
**The remapping behavior of a remapping neuron with retinal preference −20°**. **(A)** shows a single step task trial in which a stimulus is flashed from 100 ms to 200 ms, and a saccade is subsequently performed with onset at 600 ms. The saccade brings the stimulus into the receptive field of the remapping neuron, which shows a remapping response that begins before the saccade onset—a pre-saccadic remapping response. **(B)** shows the response of the same remapping neuron in a stimulus control task trial, in which a stimulus is flashed from 100 ms to 200 ms, but there is no saccade. The retinal location of the stimulus is the same as the pre-saccadic location of the stimulus in the single step task trial. In this case the remapping neuron does not respond. **(C)** shows the response of the same remapping neuron in a saccade control task trial, in which there is no stimulus present and a saccade is performed with onset at 100 ms. The saccade performed is the same as that performed in the single step task trial. Again, the remapping neuron shows no response. These three different kinds of task trial confirm that the presence of both the stimulus and the saccade are needed to trigger a response in the remapping neuron.

Figure 6 shows the remapping behavior of a remapping neuron with retinal preference −20°. It is informative to analyse the remapping behavior of the remapping neuron by inspecting its corresponding single step task trial (Figure 6A), stimulus control task trial (Figure 6B) and saccade control task trial (Figure 6C). Recall that the latter two task trials isolate either the visual or saccadic component of the single step task trial to which they correspond, and that discharge in either of these two task trials indicates that discharge in the single step task trial cannot be solely attributed to remapping.

In the single step task trial, the visual stimulus was briefly flashed pre-saccadically for 100 ms at head-centered, and therefore also retinal, location −5°. Exactly 400 ms after stimulus offset a saccade of 15° was initiated, bringing the given remapping neuron, with a retinal receptive field at −20°, over the head-centered location of the extinguished stimulus. Notice that the stimulus was never presented close to the classical receptive field location of the given remapping neuron.

Figure 6A shows that the given remapping neuron did indeed respond during the single step task trial despite the visual stimulus never being present in the receptive field of the neuron. However, when looking at the corresponding stimulus control task trial where the stimulus was flashed in retinal location −5° (Figure 6B), and the saccade control task trial where the saccade of 15° was executed (Figure 6C), there was no discharge. These results confirm that the given remapping neuron displayed genuine remapping activity during the single step task trial. Moreover, the response in the remapping neuron began well in advance of the saccade onset. Hence this negative onset latency shows that the neuron performed predictive remapping.

**Figure 7:**
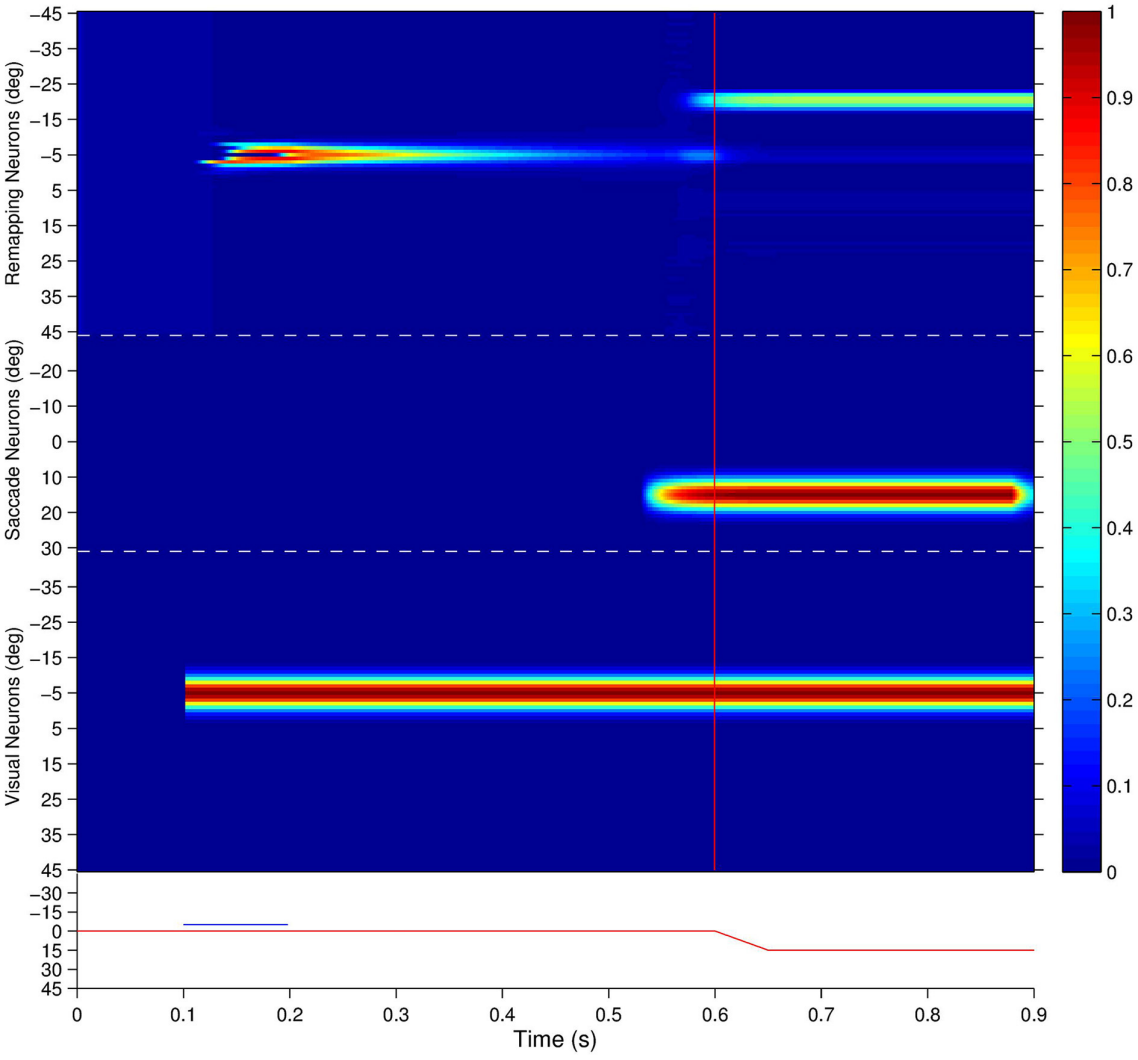
**The population responses of neurons in the trained network during a single step task trial where the stimulus is flashed at retinal location −5° and the saccade of 15° is executed 400 ms later**. This leads to a post-saccadic stimulus location of −20°. The figure shows the responses of all neurons in the remapping population (top), saccade population (middle), and visual population (bottom). Each population subplot arranges the neurons topographically along the ordinate in terms of their assigned retinal or saccade preference, α_*i*_ and β_*i*_, respectively. The traces at the bottom show the eye position (red) and retinal location of the stimulus (blue) for the trial, and the red vertical bar designates the saccade onset. It is evident that remapping neurons with preferences for retinal locations near −20° show pre-saccadic remapping in anticipation of the visual stimulus shifting into their receptive fields after the saccade.

Figure 7 shows the population responses of neurons in a flashed stimulus single step task trial. Prior to 100 ms, the visual, saccade and remapping populations were all quiescent. At 100 ms, a visual stimulus was introduced and activity immediately developed in the visual population representing the pre-saccadic retinal location of the stimulus at −5°. Next, a subset of remapping neurons with similar retinal location preferences began to respond with varying delays after the stimulus onset. At 200 ms the visual stimulus was removed and activity among the remapping neurons representing the pre-saccadic stimulus location started to decay rapidly. At 500 ms a subset of neurons in the saccade population began to represent the impending saccade of 15°. The combined activity in the visual and saccade populations then activated the corresponding combination neurons (not shown), which then stimulated a subset of neurons in the remapping population representing the upcoming post-saccadic stimulus location of −20°. These remapping neurons were activated before the saccade onset at 600 ms, and thus demonstrated pre-saccadic remapping.

Interestingly, the onset of activity in the saccade population, corresponding to the impending saccade, also caused a small level of activity to restart at the pre-saccadic retinal location of the extinguished stimulus in the remapping population. This was due to corresponding projections from combination neurons representing a combination of the pre-saccadic stimulus location and impending saccade to remapping neurons representing the same pre-saccadic stimulus location, which had been strengthened by a brief period of associative learning during training. This subtle effect is thus a specific prediction of the self-organizing hypothesis explored here.

Activity in the visual, saccade and remapping populations remained in equilibrium until the end of the single step task trial at 900 ms. At the end of the trial, the visual and saccade input populations began to go silent, and consequently so did the remapping population.

**Figure 8:**
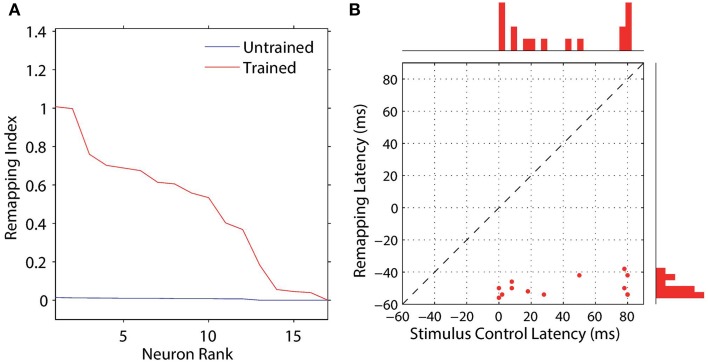
**Population analysis of the remapping responses**. Each remapping neuron is individually tested on a single step task trial that brings the visual stimulus into the receptive field of the neuron, as well as a corresponding stimulus control task trial and saccade control task trial. These tasks are then used to compute the remapping index for that neuron. **(A)** shows the remapping index for each remapping neuron. Results are shown for the model before (red) and after (blue) training. It can be seen that most of the remapping neurons have much larger remapping indices after training. **(B)** shows the remapping latency and stimulus control latency for each of the remapping neurons after training. All of the remapping neurons plotted were below the unit diagonal (dashed line), and thus had a longer stimulus control latency than remapping latency. These neurons thus performed predictive remapping. Furthermore, all of the remapping neurons had a negative remapping latency, and so were also performing pre-saccadic remapping.

We analyzed the impact of training on the remapping indices and latencies of the 17 remapping neurons that had retinal preferences α^R^_*i*_ corresponding to the post-saccadic stimulus locations of the 17 training trials. The results are shown in Figure 8 and key population metrics are summarized in Table 2. Figure 8A shows the remapping indices of the remapping neurons before training and after training. The remapping indices of neurons in the trained model were mostly much larger than for the untrained model, indicating that there was significant remapping activity only in the trained model. The average remapping index in the trained model was 0.484, while in the untrained model it was 0.0164. Figure 8B compares the remapping latency with the stimulus control latency for remapping neurons in the trained model. Due to the absence of sufficient remapping activity, no remapping latency could be decoded for the untrained model. However, for the trained model the average remapping latency among the 13 neurons for which it could be decoded was −49 ms, and all neurons were found to remap both predictively and pre-saccadically. A remapping latency could not be decoded for the four remaining neurons because they had extremely weak remapping activity, as also seen in the last four ranks for Figure 8A. In summary, remapping activity was found only in the trained model, and a range of response latencies were found that were pre-saccadic.

**Table 2:** **Population summary statistics of the response properties of remapping neurons in the model before training and after training during single step task trials**.

**Experiment 3.1**		
	**Untrained**	**Trained**
Average remapping latency	N/A (0/17)	−49 ms (13/17)
Average remapping index	0.0164	0.484
Predictive remapping	0 (0%)	13 (76.5%)
Pre-saccadc remapping	0 (0%)	13 (76.5%)

Results for the untrained model are shown in the left column, while results for the trained model are shown in the right column. Each row corresponds to a different performance metric: Average remapping latency, average remapping index, number of neurons that performed predictive remapping, and the number of neurons that performed pre-saccadic remapping. The average remapping latency is given to the closest millisecond, and the number of neurons for which latency could be computed is given.

### 3.2. Perisaccadic responsiveness shift

The classic experimental study of Kusunoki and Goldberg (2003) investigated the time course of the responsiveness of LIP neurons when visual dot stimuli were presented in the receptive fields of these neurons around the time of a saccade. In particular, they sought to characterize precisely how much an LIP neuron responded as a function of when the dot stimulus was briefly flashed with respect to a given saccade. The stimulus was flashed in either the pre-saccadic or post-saccadic head-centered location of the receptive field, and trials of each kind were referred to as *current receptive field* and *future receptive field* trials, respectively. They found that the response of LIP neurons to a stimulus in the current receptive field *decreased* as the stimulus was flashed later with respect to the saccade, and conversely that the response in the future receptive field *increased* as the stimulus was flashed later with respect to the saccade.

In the experiment described next it was attempted to confirm that the self-organizing model presented in Section 3.1 also displayed the above experimentally observed behavior, and to investigate the mechanisms by which the model achieved this. The trained model was tested on a set of delayed stimulus flash task trials, as described in Section 2.8, corresponding to the single step saccade trials the model had been trained on. The responses of the remapping neurons were analyzed using a 300 ms window aligned at 50 ms after stimulus onset, just as in Kusunoki and Goldberg (2003).

**Figure 9:**
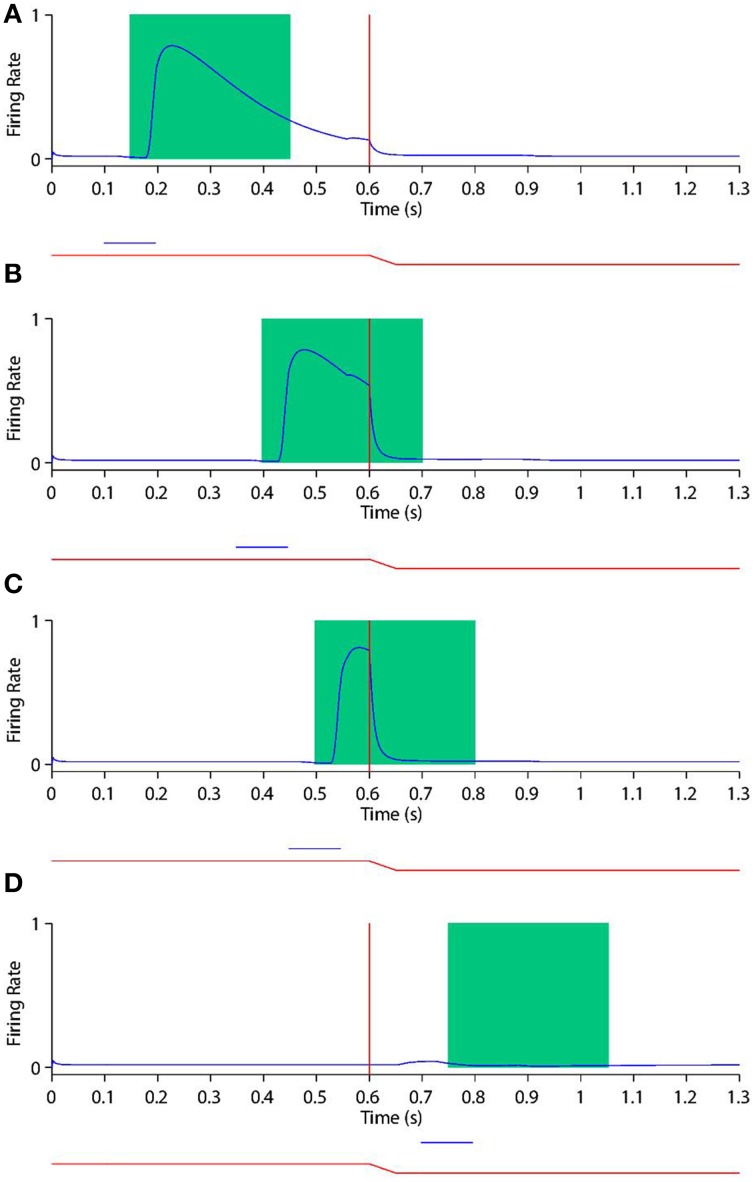
**Peri-saccadic shift in the receptive field sensitivity of a remapping neuron as the stimulus onset time is varied during current receptive field trials**. In current receptive field trials the stimulus is presented in the pre-saccadic head-centered receptive field location. Each plot shows the response of the remapping neuron with a receptive field at −20° during a current receptive field trial where the stimulus onset is at one of the the following times: 100 ms **(A)**, 350 ms **(B)**, 450 ms **(C)**, and 700 ms **(D)**. In each trial the saccade onset is at 600 ms. The two traces below the abscissa indicate the eye position (red) and stimulus presence (blue). The green rectangle shows the stimulus onset-aligned response analysis window. When analysing the neuronal response in the stimulus onset-aligned window, it is evident that the response of this remapping neuron to a stimulus presented in its pre-saccadic receptive field decreases as the stimulus is flashed later with respect to the saccade.

**Figure 10:**
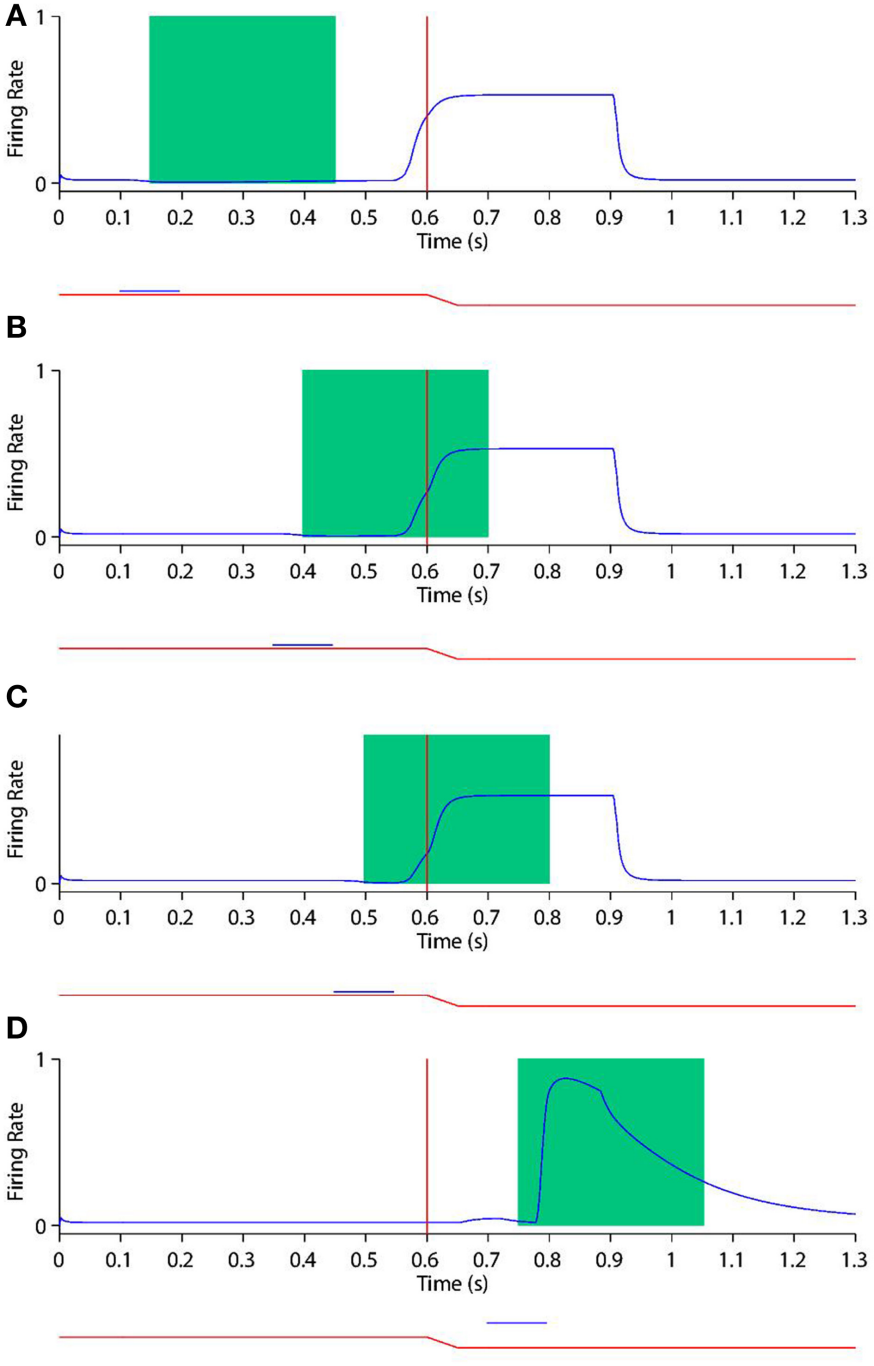
**Peri-saccadic shift in the receptive field sensitivity of a remapping neuron as the stimulus onset time is varied during future receptive field trials**. In future receptive field trials the stimulus is presented in the post-saccadic head-centered receptive field location. Conventions as in Figure 9. It is evident that the response of this remapping neuron to a stimulus presented in its post-saccadic receptive field increases as the stimulus is flashed later with respect to the saccade.

Looking at the responses of the remapping neuron with a receptive field at −20° in both current receptive field trials (Figure 9) and future receptive field trials (Figure 10) for four different stimulus onset times provides a good example for understanding the peri-saccadic shift in receptive field sensitivity of LIP neurons.

First, consider the current receptive field trials shown in Figure 9, where the visual stimulus is presented in the pre-saccadic head-centered receptive field location of the remapping neuron. When the stimulus onset is well before the saccade (Figure 9A), then the stimulus aligned window captures the majority of the response. Furthermore, the response captured by the window is not truncated by the impending saccade, which is beyond the window. So the response in the stimulus aligned window is maximal at this point. As the stimulus onset approaches saccade onset (Figures 9B,C), a longer interval of the response of the neuron is truncated by the saccade. In particular, larger portions ot the stimulus onset window capture the response truncation effect of the saccade, thus leading to a reduced response within the window. When the stimulus onset is after saccade onset (Figure 9D) there is of course no response by the neuron at all, given that the neuron's receptive field has been removed from the pre-saccadic head-centered location where the stimulus is presented. These simulation results show that the neuronal response in the stimulus onset aligned window decreases as the stimulus is flashed later with respect to the saccade.

Secondly, consider the future receptive field trials shown in Figure 10. So long as the stimulus onset is prior to saccade onset, the neuron responds more or less invariantly in terms of the magnitude and time course of the response around the time of saccade onset. So if the stimulus onset is well in advance of the saccade onset (Figure 10A), then the stimulus onset aligned analysis window does not capture any of the response, and thus registers a minimal response. With later stimulus onset times the stimulus aligned analysis window captures ever larger portions of the remapping activity, thus registering a larger response. As the stimulus onset occurs after saccade onset (Figure 10D), the response of the neuron now becomes a pure visual onset response with accompanying decay. This causes the stimulus onset aligned window to capture the majority of the strong neuronal response regardless of the stimulus onset time, thus registering a maximal response here. These simulation results show that the response in the stimulus onset aligned window increases as the stimulus is flashed later with respect to the saccade.

**Figure 11:**
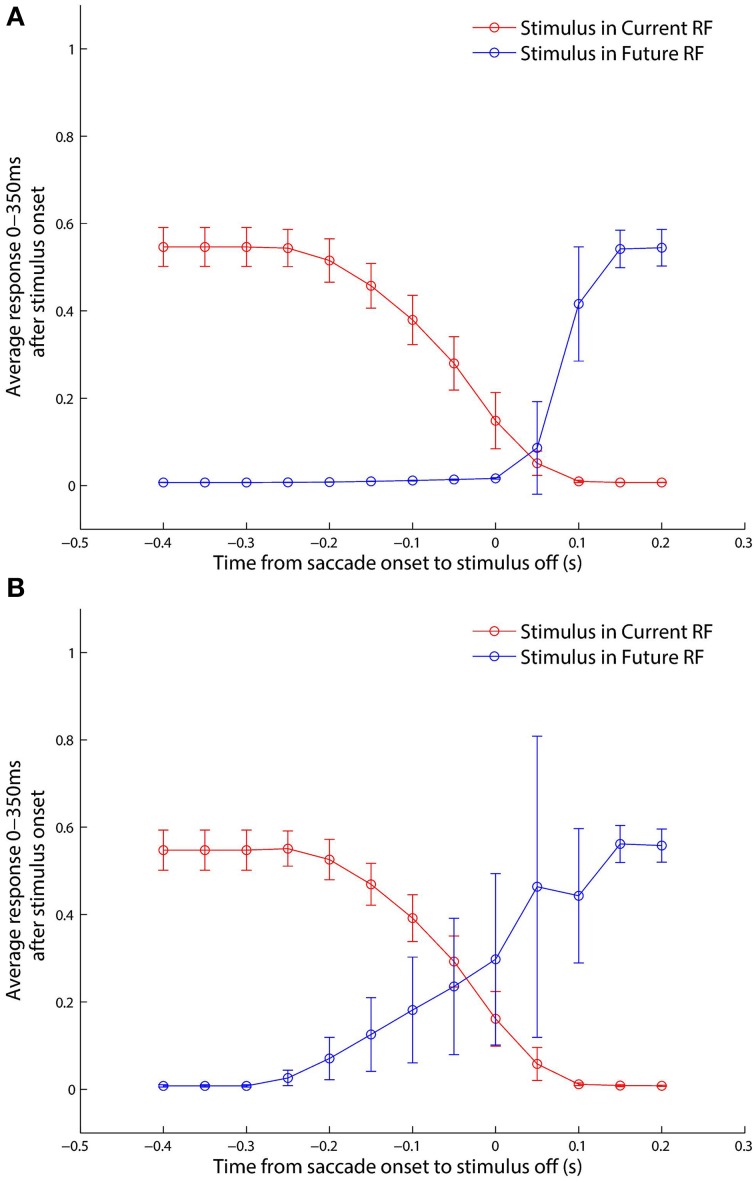
**Population analysis of peri-saccadic shift in the receptive field sensitivity of remapping neurons, before training (A) and after training (B), in current and future receptive field trials**. The plots show the average response across the 17 remapping neurons as a function of the time from the saccade onset to the stimulus extinction. The error bars represent the standard deviations.

To compare these findings with the population level analysis in Kusunoki and Goldberg (2003), all task trials of a given type (current or future receptive field) and stimulus onset time were grouped. For each such group, the average response within a stimulus onset aligned analysis window was computed across the 17 neurons. Figure 11 shows these results before and after training. The current and future receptive field trials, both before and after training, were in agreement with the experimental observations of Kusunoki and Goldberg (2003). That is, the response of the remapping neurons to a stimulus in the current receptive field decreased as the stimulus was flashed later with respect to the saccade, while response in the future receptive field increased as the stimulus was flashed later with respect to the saccade.

Interestingly, training had negligible influence on the current receptive field trial curve. This should be expected because the remapping neurons representing the pre-saccadic receptive field were being driven directly by the external visual signal during the time course of the current receptive field trials. These neurons initially responded to the stimulus, which was followed by a truncation of their responses at the saccade. Because these neuronal dynamics were driven directly by the external visual signal, they were not affected by training of the network.

Training did have a clear effect on the future receptive field trial curve. This was because the remapping neurons representing the post-saccadic stimulus location were driven by both the feedforward synaptic connections within the network, which are modified during training, as well as the direct external visual signal during the timecourse of future receptive field trials. The average response increased with increasing stimulus onset time. The initial responses corresponded to onset times where the analysis window was too early to capture any remapping activity among the remapping neurons. However, the monotonic increase reflected the fact that larger and larger portions of the analysis window were being filled by remapping activity in the future receptive field around the time of the saccade. The responses at the latest stimulus onset times reached a saturated maximum, where the analysis window simply captured the visual onset activity of the remapping neurons. These findings were in perfect argreement with the experimental observations reported in Kusunoki and Goldberg (2003).

In summary, the decreasing responsiveness of remapping neurons as the stimulus onset time occurs later with respect to the saaccade in current receptive field trials can be attributed to saccade onset aligned activity truncation, and not to any change in the feedforward visual sensitivtiy of remapping neurons after training. Likewise, the increasing responsiveness of remapping neurons in future receptive field trials can be attributed to the remapping activity displayed by these neurons.

### 3.3. Remapping from multiple directions

In Heiser et al. (2005) the authors attempted, for the first time, to comprehensively investigate the spatial characteristics of remapping in LIP. They investigated whether and how individual LIP neurons remapped activity into their receptive field from multiple directions. This was done by measuring the remapping activity of a single neuron in single step tasks requiring a saccade in each of the four cardinal directions with respect to the neuron's receptive field, where each trial relocated the receptive field to the head-centered location of a recently extinguished visual stimulus. Determining the spatial characteristics of remapping was particularly important to resolve, as it had a significant bearing on whether LIP, and other similar areas, could be supporting perceptual spatial constancy as many authors had suggested.

Importantly, the experiment did not dissociate the saccade direction from retinal location since only a single location in a given cardinal direction was examined for remapping activity. In particular, the saccades in the four cardinal directions were all of the same retinal distance. However, it seems unlikely that remapping activity in LIP neurons should be restricted to saccades in cardinal directions that are all of equal distance from the neuron's receptive field. Therefore, the model simulations described below investigated remapping activity when saccades were performed over different retinal distances. Though the saccades were peformed in only two cardinal directions because the retinal space simulated in the model was one dimensional.

**Figure 12:**
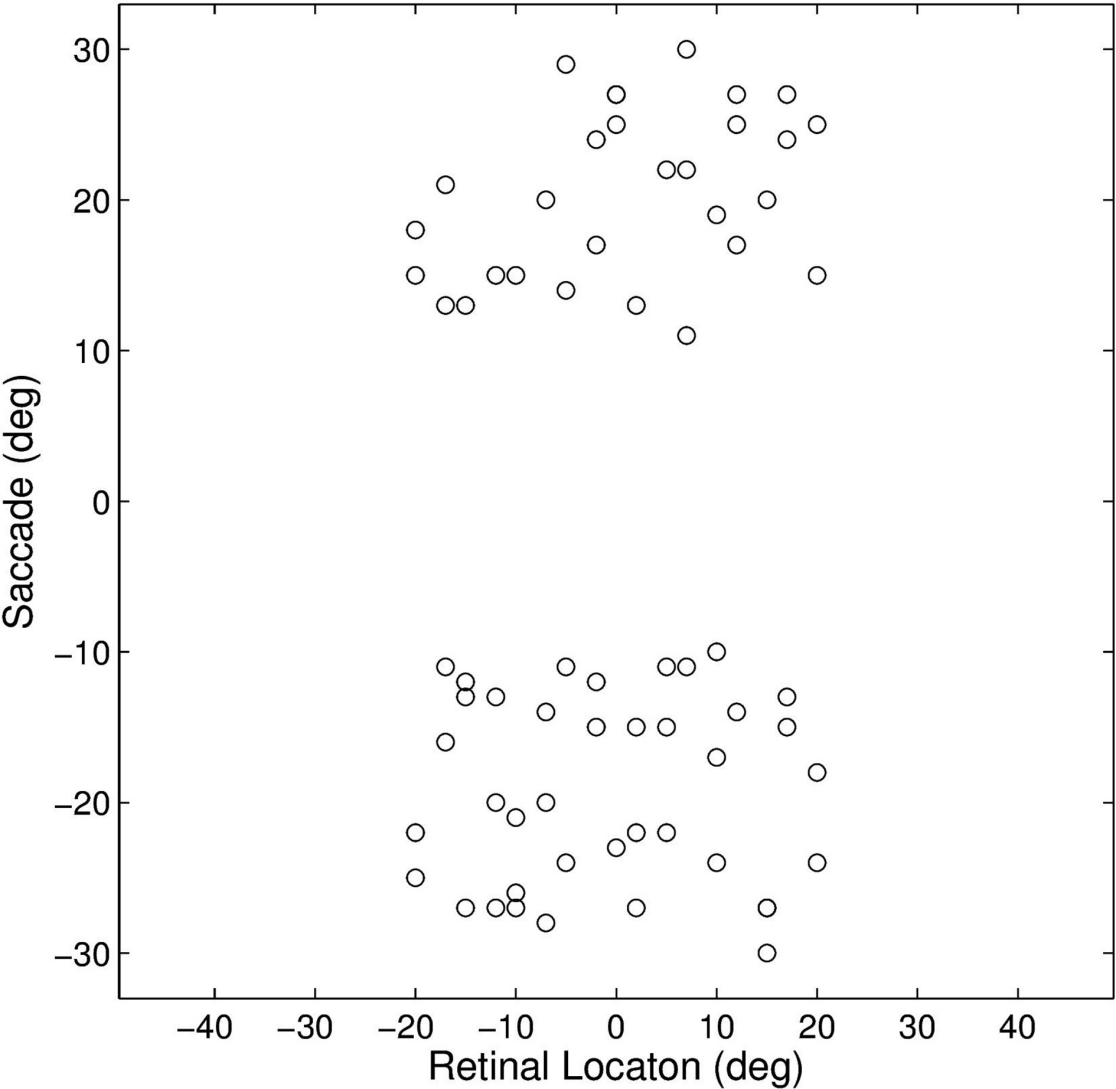
**Simulations in which individual remapping neurons are trained to remap activity from multiple (four) pre-saccadic stimulus locations**. The scatterplot shows all of the training trials in terms of the pre-saccadic retinal location of the stimulus and the corresponding saccade.

In the previous Experiment 3.1 there where 17 training trials, where each trial corresponded to one particular combination of pre-saccadic stimulus location, saccade, and resulting post-saccadic stimulus location. This set up the synaptic connections to enable the remapping neurons representing the post-saccadic stimulus location to exhibit remapping activity in response to a stimulus presented in the trained pre-saccadic location followed by the corresponding saccade. In the experiment described next there were four different training trials associated with each of the same original 17 post-saccadic stimulus locations, each with a different combination of random pre-saccadic stimulus location and corresponding saccade. This made the total number of training trials 17× 4 = 68. Figure 12 shows all training trials in terms of the pre-saccadic retinal location of the stimulus and the corresponding saccade. The model was trained for 20 epochs with the same parameters as before, except that ϕ^C^ was varied because this parameter influenced the likelihood of a given combination neuron driving a particular remapping neuron.

**Figure 13:**
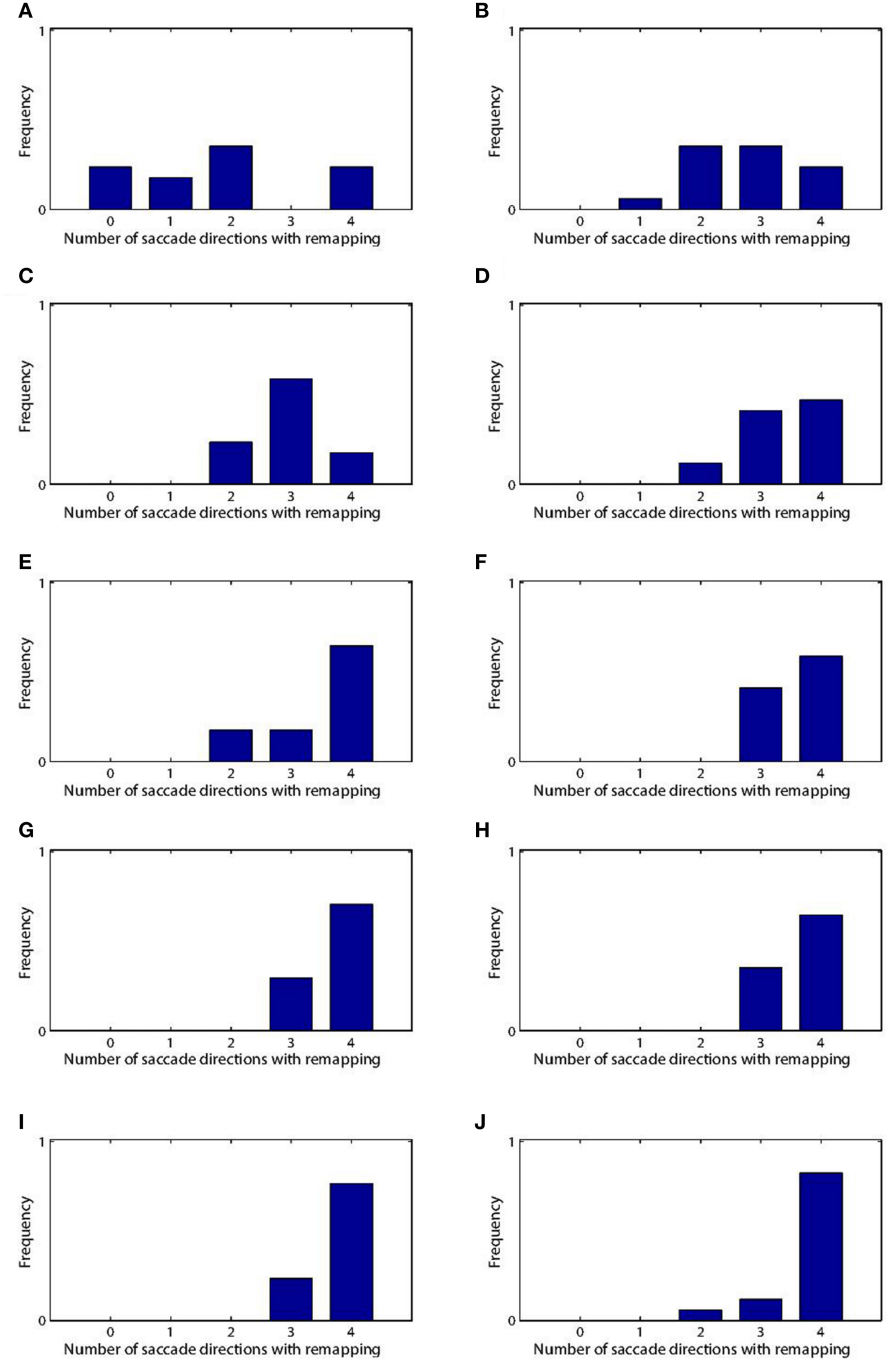
**Frequency distributions showing the proportion of the 17 selected remapping neurons that learned to remap activity from 0, 1, 2, 3 or 4 pre-saccadic stimulus locations**. Each distribution corresponds to a simulation with a particular value of ϕ^C^, which is incremented in steps of 0.1 from 0.1 **(A)** to 1.0 **(J)**. A neuron was classified as remapping from a given pre-saccadic retinal location if the corresponding remapping index was greater than zero and increased from the untrained network.

Given the training and testing trials described above, a given remapping neuron could remap activity anywhere from 0 to 4 pre-saccadic stimulus locations. A neuron was classified as being able to remap a given pre-saccadic stimulus location if the remapping index from the single step trial was greater than zero and larger than the remapping index from the same trial prior to training. Frequency distributions showing the proportion of the 17 selected remapping neurons that learned to remap activity from 0, 1, 2, 3 or 4 pre-saccadic stimulus locations are shown in Figure 13. It can be seen that for all values of ϕ^C^ there were at least some remapping neurons that were able to remap activity from all four pre-saccadic stimulus locations. However, as ϕ^C^ was increased from 0.1 to 1.0, the latter of which being the standard value, the neurons tended to remap from an increasing number of pre-saccadic retinal locations. The parameter ϕ^C^ controlled how well-connected a neuron in the remapping population was with neurons in the combination population. Hence, an increase in this parameter made it more likely that a given remapping neuron trained on a particular single step trial would be connected with combination neurons representing the corresponding pre-saccadic stimulus location and saccade. So increasing ϕ^C^ made it possible for a remapping neuron to remap from more pre-sacadic stimulus locations.

In summary, these results show that remapping neurons in the model were able to remap from multiple pre-saccadic stimulus locations, which was in agreement with the findings of Heiser et al. (2005). Furthermore, the population distribution of remapping neurons that could remap activity from a number of different pre-saccadic stimulus locations became more skewed toward a larger number of locations as the connectivity rate between the remapping and combination populations was increased.

## 4. Discussion

### 4.1. Main findings

This paper investigated the feasibility of the hypothesis, described in Section 2.1, that a biologically plausible process of visually-guided learning could produce neurons with a range of experimentally observed perisaccadic receptive field properties. The core hypothesis assumed that, since primates move their eyes more rapidly than the head, visual stimuli tend to remain stationary in the reference frame of the head during saccades. Some visual neurons will encode the retinal pre-saccadic location of a stimulus, while other saccade neurons encode the retinal target location of the saccade. These neurons may in fact be distributed across a number of different areas of the primate brain. Efferent projections from these two types of neuron may then converge on a population, or perhaps multiple populations, of combination neurons. These neurons learn to respond to specific combinations of pre-saccadic stimulus location and saccade target location. It was hypothesized that some visual neurons may have significantly delayed responses to the presence of stimuli due to the time taken for signals to propagate across successive layers of the visual system. Moreover, some visual neurons may maintain their activity for some time after a stimulus is extinguished by the operation of local recurrent circuits leading to attractor states. These two mechanisms will ensure that some visual neurons representing the retinal pre-saccadic stimulus location will remain active for a short time after the saccade. This delayed response will be passed on to the combination neurons, which will also remain active for a short time after the saccade. The network may then learn an association from those combination neurons representing a combination of the pre-saccadic stimulus location and saccade target location to those remapping neurons representing the post-saccadic stimulus location. After this process of visually-guided learning, the subsequent presence of a visual stimulus in a pre-saccadic location combined with the execution of a saccade will activate the corresponding combination neurons, which will in turn activate the remapping neurons that represent the corresponding post-saccadic stimulus location. This remapping process may still occur even if the visual stimulus is extinguished before the saccade. This basic hypothesis is very general and could be instantiated in many different forms of network architecture with different numbers of layers and patterns of connectivity between these layers. The governing neuronal and synaptic dynamics could also be varied considerably, yet still embody the same core computational principles outlined here. In this paper, we have shown the successful operation of these principles in the simplest biologically plausible network architecture shown in Figure 1. This highly idealized network architecture is not intended to be mapped directly onto specific areas of the primate visual system, although we have suggested some loose correspondences based on the observed firing characteristics of neurons in these areas. The model shown in Figure 1 was able to learn to display the phenomena of predictive remapping, pre-saccadic remapping, trace remapping, responsiveness shift and spatially independent remapping.

The self-organizing model shown in Figure 1, initially set up with random synaptic connectivity and weighting, was exposed to a series of visual stimuli that were stationary in head-centered space across saccades. Around the time of saccade onset the model learned to associate the pre-saccadic retinal location of the stimulus and the associated saccade with the corresponding post-saccadic retinal location of the stimulus. Through computer simulation, a number of experimentally observed response properties were found to have developed. After training, the model could remap activity into the receptive field of remapping neurons corresponding to the post-saccadic retinal location of the stimulus, if the appropriate combinaton of pre-saccadic stimulus location and saccade occurred. This remapping occurred even though the visual stimulus had been extinguished well in advance of the saccade which would bring the neuron's retinal receptive field over the head-centered location of the stimulus. This was because the remapping was being driven by a transient encoding of the visual scene in a retinal frame of reference. This explains the classic result of Goldberg and Bruce (1990) and also Duhamel et al. (1992), which found that even a stimulus that had been extinguished as long as 1 s prior to saccade onset would excite a neuron if its receptive field was brought over the head-centered location previously occupied by the stimulus. Moreover, it was found that the remapping neurons in the model would start responding with a much lower latency compared to a visual onset trial. Indeed, most neurons responded even in advance of the saccade onset, resulting in predictive remapping, which was also observed by Duhamel et al. (1992). This was because the saccade population in the model began encoding the impending saccade in advance. Hence, the coincidence of the encoded saccade and transiently encoded visual scene excited the combination population, which in turn stimulated the remapping neurons, before the saccade had been initiated. A range of response latencies was observed in the model, as has been observed in LIP (Duhamel et al., 1992), FEF (Umeno and Goldberg, 1997), and SC (Walker et al., 1995). However, there was a population bias toward predictiveness which was not reflected in experimental observations. This could, however, be ameliorated by introducing sufficient variability in the Δ^S^_PRE_ parameter across neurons, which had been kept uniform at 70 ms in all experiments.

The timecourses of remapping dynamics around the time of a saccade were tested in the same manner as in Nakamura and Colby (2000) and Kusunoki and Goldberg (2003). The population results found in the model agreed qualitatively very well with the experimental results from LIP, and also from visual cortical areas V3A, V3, V2, and V1. These result have been interpreted as a shift in the responsiveness in the current and future receptive field locations around the time of the saccade. Specifically, the responses of neurons to a stimulus in the current receptive field decrease as the stimulus is flashed later with respect to the saccade, while the responses in the future receptive field increase as the stimulus is flashed later with respect to the saccade. The model examined here explains the data with more clarity. First consider the responses of remapping neurons with their receptive field located on the stimulus pre-saccadically, i.e., the current receptive field trials, and the corresponding population curves shown in Figure 11 as red plots. With a stimulus aligned analysis window, the decrease in the neuronal responses emerges as a result of the increasing portions of the analysis window passing the onset time of the saccade, and therefore being void of activity, despite the fact that the timecourse of responses prior to this point is always the same. Second consider the response of remapping neurons with their receptive fields located on the stimulus post-saccadically, i.e., the future receptive field trials, and the corresponding population curves shown in Figure 11 as blue plots. With a stimulus aligned anlaysis window, the increase in the neuronal responses emerges as a result of the increasing portion of the analysis window passing the onset time of the saccade, and therefore capturing the remapping activity. However, neither of these scenarios actually requires any change to the timecourse of neuronal responses prior to saccade onset, and hence are perhaps not best described as a change in responsiveness leading up to the saccade. Instead, the apparent change in responsiveness is due to the interaction of saccade onset aligned events such as response truncation with the relatively long analysis windows.

The spatial properties of remapping were tested in a similar manner to Heiser et al. (2005), where they examined the remapping of stimuli located 20° from the receptive field along one of the four cardinal directions. The experimental results of Heiser et al. (2005) were presented as being about the effects of saccade direction. However, their findings are perhaps better understood as being about pre-saccadic stimulus location. When a neuron was found to remap stimuli only in a single cardinal direction, it was classified as unidrectional. However, it is possible that the neuron would have remapped stimuli at other untested distances along the three other cardinal directions, or also along other non-cardinal directions. Likewise, it was quite possible that the neuron would not have remapped stimuli at all other distances along the cardinal direction for which it did display remapping. Hence, their experimental results are best interpreted in a more general way as about remapping from different stimulus locations. Indeed, although the model tested in this paper could develop some remapping neurons that failed to learn to remap from all four pre-saccadic locations that they were trained on, this effect was quite stochastic and would be shaped by random factors such as the diluted connectivity and initial strengths of the afferent connections to the combination neurons. In other words, with regular unbiased training, there was no systematic way in which remapping neurons could learn a regular bias in terms of their selectivity to remapping from four cardinal directions as tested by Heiser et al. (2005). As a result, the model was examined for remapping from four random directions and distances. The key result from the original paper of Heiser et al. (2005) was replicated in the model. Many remapping neurons remapped in multiple directions. However, there was substantial variability among remapping neurons which depended critically on the rate of connectivity between the combination population and remapping population. Lower connectivity caused remapping neurons to learn to remap in fewer directions, as expected.

### 4.2. Previous models

A range of models have previously been proposed to explain various remapping phenomena that the self-organizing model presented here has sought to account for. Some of these earlier modeling studies are described next.

The early work of Droulez and Berthoz (1991) showed that an activity packet could be moved within a topographic population of neurons by an external eye velocity signal. Their model shared some architectual similarities to the work presented here. However, it required topographic organization of the neural population, something which is not prevalent in LIP (Blatt et al., 1990). Their model also predicted that activity would move continuously through the neural population, guided by an eye velocity signal. However, the model is not compatible with predictive and pre-saccadic remapping, nor with the variability in remapping latency across neurons. Lastly, the circuit elements in the model switch between being excitatory or inhibitory depending on the eye velocity input, something which is not thought to be possible in biological neurons (O'Donohue et al., 1985).

The work of Krommenhoek et al. (1993), Mitchell and Zipser (2001), and White and Snyder (2004) represent classic examples of models organized through biologically implausible processes of supervised error correction learning to produce the correct motor error for double-step saccade tasks. The two models of Krommenhoek et al. (1993) required eye position at the time of target selection as an explicit input signal, and were able to combine this with the current eye position and retinal target location to produce the correct motor error. It does, however, seem unclear why there should be an explicit mechanism for saving eye position at the initation of double-step saccades given the highly unrealistic nature of the task. The model of Mitchell and Zipser (2001) was a shifting packet model, much in the spirit of Droulez and Berthoz (1991), which was found to be able to produce outputs in both eye-centered and head-centered reference frames. The model of White and Snyder (2004) also had the same basic structure. However, it was trained to remap gaze fixed stimuli when a separate reference frame cue was turned on without an adequate explanation for where such a cue might actually derive from in the brain. These three models thus offered no plausible mechanism by which they could self-organize their synaptic connectivity.

The work of Quaia et al. (1998) is most similar to the model developed here. It had a very similar basic architecture, where a module in LIP was responsible for shifting activity between different parts of a retinal population in LIP, based on the activity in a population of FEF neurons encoding impending saccades. The model was hardwired to produce the correct remap. Like the model developed here, it also had a saccade aligned damping signal that produced response truncation. However, in contrast, it produced a range of remapping latencies by having remapping activity flow back and forth between phasic neurons in FEF and LIP. While the authors do suggest that some of the primary circuitry could self-organize in a similar manner to the model presented here, it still has some important shortcomings. First, its function depends on very detailed dendritic operations and wiring that could not be accounted for by self-organization. Second, the model depends on topographically aligned nesting between LIP and FEF, which is not likely to be possible given that LIP has very weak topography (Blatt et al., 1990).

### 4.3. Future work

As discussed above, the network architecture simulated in this paper and shown in Figure 1 is the simplest architecture capable of displaying the computational mechanisms hypothesized in Section 2.1. However, we anticipate that in the brain these mechanisms may be distributed across several interacting layers, with a more complex pattern of synaptic connectivity between and within layers. Indeed, each of the four different types of neuron shown in Figure 1 may also be distributed across multiple layers. The hypothesized learning mechanisms should continue to operate under these more complex, and potentially more realistic, conditions. So future modeling work will investigate how the learning mechanisms hypothesized in this paper could be instantiated across the known visual areas of the primate brain. This will involve developing more detailed multi-layer network models incorporating known brain areas, as well as the known connectivity between and within these areas. Furthermore, the equations governing the neuronal dynamics of the model investigated in this paper incorporated terms that were explicitly dependent on the time of the stimulus onset and saccade onset. These terms were used to model specific biological processes described in Section 2.9 at a rather abstract level. However, in future work, we will aim to replace these high-level model formulations with neuronal equations that do not incorporate such explicit dependencies on stimulus onset and saccade onset. Instead, the required neuronal dynamics, such as signal transmission delays and attractor states used to keep the visual neurons active for a short period after the saccade, will emerge naturally from the network architecture itself.

Another question which should be addressed in future work is how well the model will self-organize when exposed to scenes with multiple simultaneously visible visual targets. Will the model be able to associate a combinations of a pre-saccadic retinal location and a saccade with the correct post-saccadic retinal location? Previous work with training regimes with multiple targets suggests that it would (Stringer and Rolls, 2000; Mender and Stringer, 2014), so long as the model is exposed to each combination in conjunction with a range of other pre-saccadic retinal stimuli locations. This is exactly what occurs with scenes with multiple simultaneously visible targets placed in uncorrelated locations. This will cause the combination neurons to decouple the given combination from all the other locations, since the combination is not correlated with any of them. As a result each combination will be represented independently, since the components of the combination itself are correlated, and therefore the remapping neurons will learn the correct combination neurons just as before.

A potentially fruitful further line of inquiry based on the initial work presented in this paper could be to attempt to interpret the range of physiological and behavioral results associated with split brain macaques performing interhemispheric stimulus remapping. It has been found that both behavioral performance during double-step saccades Berman et al. (2005) and interhemispheric remapping in such trials (Heiser et al., 2005) is influenced by commissurotomizing the forebrain commissures, and that the two are related (Berman et al., 2007). Moreover, improvement in the performance of these tasks after initial impairment was found, and neural plasticity was thought to be a primary candidate for this improvement (Berman et al., 2005). This could potentially be modeled by commissurotomizing the model and attempting to retrain it in accordance with the behavioral protocol. Another issue this model could help shed light on is how multiple interconnected areas, such as LIP, FEF, and SC, interact in producing their remapping responses. It is not known whether each area independently generates its own remapping, or whether it is a distributed process.

## Funding

This work was funded by The Oxford Foundation for Theoretical Neuroscience and Artificial Intelligence and The Norway Scholarship.

### Conflict of interest statement

The authors declare that the research was conducted in the absence of any commercial or financial relationships that could be construed as a potential conflict of interest.
